# Effects of soil nutrient enrichment on biomass, herbivores, and their predators differ between tree species in the Brazilian Cerrado

**DOI:** 10.1007/s00442-026-05863-z

**Published:** 2026-02-07

**Authors:** Carla Faleiro Tinoco, Sílvia Castro, Rodrigo Damasco Daud, Vanessa Leonel Falchi, Júlia Almeida Reis, Stefany Ribeiro Constantino, Carlos de Melo e Silva-Neto, Luísa Gigante Carvalheiro

**Affiliations:** 1 Departamento de Ecologia, Instituto de Ciências Biológicas, Universidade Federal de Goiás (UFG), Av. Esperança, s/n, Campus Samambaia (Campus II), Goiânia, GO 74690-900 Brazil; 2 Centre for Functional Ecology, Associate Laboratory TERRA, Department of Life Sciences, University of Coimbra, Calçada Martim de Freitas, 3000-456 Coimbra, Portugal; 3 Laboratório de Taxonomia, Ecologia e Interações de Aracnídeos (TEIA), Departamento de Ecologia, Instituto de Ciências Biológicas, Universidade Federal de Goiás, Goiânia, GO 74690-900 Brazil; 4 Programa de Pós-Graduação em Biodiversidade Animal, Instituto de Ciências Biológicas, Universidade Federal de Goiás, Goiânia, GO 74690-900 Brazil; 5 Instituto Federal de Educação, Ciência e Tecnologia de Goiás, Centro de Referência em Pesquisa e Inovação - IFG, R. Dona Sanduca, Sítio de Recreio Ipê, Goiânia, GO 74594-111 Brazil; 6 Universidade Estadual de Goiás, Unidade Ipameri, Setor Universitário, Ipameri, GO, CEP 75780-000 Brazil

**Keywords:** Plant–herbivore interaction, Phytophagous mites, Fertilization effects, Soil nutrient enrichment, Trees and shrubs

## Abstract

Human-induced changes in nitrogen (N) and phosphorus (P) global cycles and availability significantly impact plant growth and nutritional composition, thereby affecting ecosystem dynamics. However, research on the effects of increased nutrient availability often focuses on plant community-level effects, overlooking interspecific variability and neglecting impacts on higher trophic levels. Using a controlled fertilization experiment with six tree species that naturally occur in the Cerrado biome (Brazilian savannas), we showed that there is substantial interspecific variation in how plants respond to nutrient changes. This reflects the existence of competitive advantage for certain native species under a scenario of increased soil nutrient availability. Such effects propagated to higher trophic levels (herbivores and their predators), also varying between plant host species. The strength and direction of N input effect depended on P levels and the type of herbivores. Large invertebrate leaf herbivores were less affected than phytophagous mites. Impacts on higher trophic levels (predatory mites) were less pronounced than on phytophagous mites. Overall, we show that ongoing soil nutrient enrichment has the potential to alter interspecific competition dynamics in plant communities with consequences for ecological interaction partners. These findings have important implications for conservation and ecosystem management, especially in areas highly exposed to soil nutrient enrichment due to farming and industrial activities.

**Supplementary Information:**

The online version contains supplementary material available at 10.1007/s00442-026-05863-z.

## Introduction

Biogeochemical flows, especially those of nitrogen (N) and phosphorous (P), are greatly affected by anthropogenic activity, affecting ecosystems worldwide (Steffen et al. 2015). While N is a limiting nutrient for plants in most environments, human-driven increases in its availability are leading to biodiversity losses and changes in ecosystem functioning (Bobbink et al. 2010). Most studies that evaluate the impacts of ongoing soil nutrient enrichment on terrestrial communities tend to focus on community-level patterns of plants (Bobbink et al. 2010; Stevens et al. 2018). However, increased N availability can also lead to a variety of bottom-up effects, influencing trophic interactions through several mechanisms (Chen et al. 2010). Such effects on plants and interacting partners likely depend on traits of the plant species that regulate how the plants make use of the existing nutrients (Barbosa et al. 2014). Despite this, only a few studies investigate the interspecific variability of these effects (e.g. Barbosa et al. 2014) and how such impacts propagate to higher trophic levels at species level (e.g., herbivores and their natural enemies; but see Chen et al. 2010; Pöyry et al. 2017; Stevens et al. 2018; David et al. 2019 for community-level analyses or reviews on propagation of impacts of nutrient enrichment). This knowledge gap is particularly pronounced in tropical regions and may limit our ability to predict the impacts of global change and plan appropriate management actions.

Savanna ecosystems, such as the Cerrado, have ancient geological formations with advanced soil weathering processes, limiting the development and productivity of its vegetation by the low availability of nutrients in the soil, mainly N and P (oligotrophic soils, Bustamante et al. 2006; Haridasan 2008). Many species in these environments have specific and energetically costly strategies that increase access to soil nutrients (e.g., root structures like root dimorphism, resorption of nutrients before leaf senescence and symbioses with microorganisms; Haridasan 2008; Oliveira et al. 2015). These strategies require a substantial investment and can, depending on plant traits (Barbosa et al. 2014), result in less investment of plants in above-ground growth (Hoffmann and Franco 2003; Lambers et al. 2020). Hence, in a scenario of increased nutrient availability, the negative impacts on plants above-ground growth may be more accentuated in these oligotrophic and highly biodiverse tropical ecosystems (e.g., Lambers et al. 2020). The change in plant nutritional quality, induced by increased N availability, may be more pronounced in soils co-limited by N and P and can affect consumers. Indeed, increased N availability tends to increase P limitation both in plants and their consumers (Vogels et al. 2023). Understanding the diversity of responses of plant trees in oligotrophic soils (e.g., Cerrado) to changes in nutrient soil levels is essential to better predict impacts of global environmental changes and to define adequate conservation and restoration practices.

Effects of increased N availability can vary between plant species depending on their nutritional requirements. Plant species adapted to N rich soils (nitrophilous) can better take advantage of such an increase than species that are adapted to oligotrophic soils (nitrophobous) (Bobbink et al. 2010; Pöyry et al. 2017; Stevens et al. 2018). N-driven changes in plant species composition (i.e., abundance, richness, evenness) will affect the availability of resources for primary consumers (e.g. leaves, Throop and Lerdau 2004; Stevens et al. 2018). Another important pathway through which soil nutrient enrichment can affect plant consumers is through changes in the nutritional quality of plant resources. Plant species naturally differ in their primary (e.g., protein and carbohydrate; Wilson et al. 2019) and secondary (e.g., alkaloids; Kessler and Kalske 2018) compound content, and such variability is known to influence primary consumers performance and fitness (Throop and Lerdau 2004; Nijssen et al. 2017; Stevens et al. 2018). The concentration of such compounds in leaves is affected by changes in soil N availability, especially when P is not a factor limiting N fixation (Throop and Lerdau 2004; Vitousek et al. 2010), changing how herbivores perceive the quality of the plant as a food resource. Indeed, several amino acids necessary for herbivore growth and reproduction are solely obtained through diet (i.e., are essential amino acids). Thus, an increase in N soil levels can change the quality and palatability of plants as a food resource (Throop and Lerdau 2004; Li et al. 2016; Stevens et al. 2018). Furthermore, increasing N supply can also significantly decrease concentrations of secondary C-based compounds (e.g., polyphenolics and phenolics), which act as herbivory defenses, reducing plant resistance to herbivory (Chen and Ni 2011; Sun et al. 2020). Consequently, such N-driven effects are likely to make the plant more susceptible to herbivory (Throop and Lerdau 2004; Li et al. 2016; Stevens et al. 2018).

The effects of changes of soil N content in plants and herbivores can also propagate to higher trophic levels, e.g., changing the susceptibility of herbivorous insects to their natural enemies (i.e., predators, parasitoids, and pathogens; Throop and Lerdau 2004) or changing the production of plant’s volatile defense compounds that act as foraging signals for natural enemies (Chen and Ni 2011). However, there is still little information on how changes in soil nutrient levels propagate from plants to herbivores and predators, particularly in tropical ecosystems.

In this work, we used a controlled fertilization experiment to investigate the impacts of soil nutrient changes on plant biomass (number of leaves), herbivory, and predation in six tree species that naturally occur in Cerrado (but also occur in other biomes). Since the experimental plants were not exposed to competition with other plants, we expected that, moderated N increases would lead to a positive effect on number of leaves, these effects being more accentuated when P is not a limiting factor (expectation 1). We also expected herbivores to prefer plants grown in soils with moderate levels of N added than plants grown in low levels of N, these effects cascading to herbivore natural enemies (expectation 2). On the other hand, above certain thresholds of N and P levels, the plant may suffer a nutritional imbalance or start to invest more in toxic compounds (Throop and Lerdau 2004; Chen et al. 2010), reducing the herbivores and their natural enemies (expectation 3).

## Materials and methods

This study was carried out in five study sites within the Campus (Samambaia) of Federal University of Goiás (Table S1), Goiânia, Goiás, Brazil. The study sites were at least 500 m apart. Due to the limited dispersal ability of mites (e.g., Jung 2005) and insect herbivores (Ricketts et al. 2008; Zurbuchen et al. 2010), this distance ensured that plants in each site were exposed to independent communities of these guilds. The sites are within a region of Brazilian Cerrado, with climate classified as Aw (Köppen), where rainfall ranges from 1600 to 1900 mm year^−1^, with an average annual temperature between 20 °C and 22 °C (Alvares et al. 2013). Besides sharing the same climatic regime, the areas exhibit a similar pattern of insolation, rainfall, and winds due to their proximity.

### Focal plant species

This study focused on six tree species that naturally occur in the Cerrado biome, being relatively common. These species also occur in other Brazilian biomes, having different spatial ranges within South America, some being more widespread than others (Table S2). The selected species were chosen considering the availability of individuals in local commercial nurseries and cover a variety of strategies of nutrient acquisition (Table S2). Four of the selected species are fast-growing: *Schinus terebinthifolia* Raddi (Anacardiaceae) and *Solanum lycocarpum* A.St.-Hil. (Solanaceae) being pioneer species and *Inga vera* subsp*. affinis* (DC.) T.D.Penn*.* and *Psidium hians* Sw. (Myrtaceae) being early secondary successional plants. *S. terebinthifolia* and *I. vera* are also known to occur in biomes where nutrients are typically not a limitation (Pampas, Pantanal, Amazonia, and Atlantic Forest, Table S2). Two species are slow-growing typical of later secondary successional stages, *Campomanesia cf adamantium* (Cambess.) O.Berg (Myrtaceae), and *Eugenia gemmiflora* O.Berg (Myrtaceae), the first occurring in biome where nutrients are typically not a limitation (Atlantic Forest, Table S2).

All *S. lycocarpum* seedlings were obtained from the sowing of seeds collected from trees in natural areas. Four seeds were added to each pot and, before the addition of fertilizer, only the largest plant was left in each pot. For the other five plant species, saplings were directly acquired from local nurseries.

A total of 730 individuals were reared in pots throughout the entire experiment. In October 2018, 350 individuals (70 individuals of *S. terebinthifolia*, *I. vera*, *E. gemmiflora*, *C. cf adamantium* and *S. lycocarpum*) were transferred to 11 L pots. The individuals of *I. vera* were planted in 18 L pots, due to its growing requirements. Due to high mortality, a further 180 individuals (45 saplings of *C. cf adamantium*, 40 of *E. gemmiflora*, 25 of *I. vera* e 40 of *S.terebinthifolia* and 30 seeds of *S. lycocarpum*) were acquired in February 2019 and 200 more individuals were added in November 2019 (50 saplings of *S.terebinthifolia*, 30 of *I. vera* and 60 seeds of *S. lycocarpum*), including a new plant species, *P. hians* (60 saplings of *P. hians*). Due to lack of availability of saplings of *C. cf adamantium* and *E. gemmiflora* in the nursery, no extra saplings of these species were included in the experiment.

The soil used in the experiment was red underground soil (oxisol) commercially purchased, which was impoverished in terms of organic matter (0.75%, i.e., 3 to 6 times less than normal levels in Cerrado, having between 2 and 5% of organic matter; Resck et al. 1991; Ruggiero et al. 2002; Lopes and Guilherme 2016). Soil P levels are like those found in preserved natural soil from the Cerrado [ca. 1.83 mg/dm^3^, which corresponds to 3.66 kg/ha, normal levels below 2 mg/dm^3^ (4 kg/ha), Lopes and Guilherme 2016].

### Experimental set-up

In each of the five study sites (Table S1) is a complete randomized block design arrangement (Figure S1), with six treatments with different levels of nutrients (see below). Each treatment contained three pots from each of the plant species (the exception being *P. hians* that due to lack of available saplings only had two pots). The position of each treatment in each study site and the order of the plants in each treatment was randomized. A drip irrigation system has been installed and regulated to provide approximately 750 ml of water per day for each sapling.

Treatments combined three different levels of N (N0 = without addition of N, N1 = 60 kg ha^−1^per application, N2 = 130 kg ha^−1^ per application) and two levels of P (P0 = without addition of P, P1 = 40 kg ha^−1^ per application). The application of N was done in the form of Urea [CO(NH_2_)_2_], and for P, we used simple superphosphate [Ca(H_2_PO_4_)_2_]. The nutrient concentration used aims to replicate the practices recommended for fertilizer use agricultural crops farms. The application of 60 kg ha^−1^of N and 40 kg ha^−1^of P corresponds to a recommended level for some of the most common crop species in the study region (i.e., common bean, see Ramos et al. 2018), and 130 kg ha^−1^ of N corresponds to an excessive level of N, but is frequently applied by farmers in Cerrado environments (Ramos et al. 2018). The amount of fertilizer (N and P) applied in each pot was calculated based on their concentration in the applied elemental form (45% of N in Urea and 21% of P in simple superphosphate for P). The nutrients were applied directly to the soil in each pot approximately every 3 months since November 2018.

### Plant vegetative metrics

Surveys to extract information on plant metrics were done in May, August 2019, and October 2020. For each individual plant, at each sampling event, we recorded the number of leaves per tree. We used the number of leaves as a proxy for plant biomass increase in response to soil fertilization (Throop and Lerdau 2004), which serves as a more direct indicator of food availability for the herbivores studied here.

When the plant was introduced in the experiment, we collected information on their height (stem length, cm). Plant height was used in the analyses of number of leaves to control for variations in size due to differences in age and planting times.

### Herbivores and higher trophic levels

Herbivory metrics considered in this study were: leaf herbivory by external large leaf-feeding invertebrate herbivores (e.g., ants, caterpillars, beetles and grasshoppers, no mammal herbivores were observed in the study areas) and density of phytophagous mites. In each sampling event, leaf herbivory was measured as the percentage of plant leaves showing signs of leaf tissue consumption by herbivores. If a leaf or leaflet was completely missing, this information was not counted as herbivory. Then, for each individual plant that had at least 12 leaves, we collected three leaves. If an individual had fewer than 12 leaves, the collection was adjusted so that we would never collect more than 25% of the total number of leaves of every individual.

To avoid choosing which leaves to collect, we decided a priori to collect the 4th, 5th and 6th leaves, counting from the apex. For species with composed leaves with large leaflets (i.e., *I. vera*), leaflets were collected. Leaves were stored in vials with ethanol 70%. Then, under laboratorial conditions, all the collected leaves were washed by shaking vigorously the recipient for 30 s to release all mites. Samples were then analyzed under stereoscopic microscope and all mites found were mounted on slides with Hoyer's medium (Moraes and Flechtmann 2008). The mites were then quantified and identified to the lowest possible taxonomic level (Moraes and Flechtmann 2008) and separated into morphogroups under phase contrast microscopy. Slides with mites were deposited in the collection of Acari of the Laboratory of Taxonomy, Ecology and Interaction of Arachnids (TEIA) at the Federal University of Goiás (UFG). The mite community was classified into three groups based on the predominant feeding habits of each group: (1) phytophagous, (2) predators and (3) undetermined/unknown (see Table S3). In the latter, we placed mites that were not identified to species level (e.g., immature or damaged individuals) and belong to families harboring species with different guilds. Groups that do not have species that feed on plants (Oribatida and Winterschimidtiidae) like mycophagous species (which made up only a very small proportion of the mites collected) were excluded from analysis.

### Data analysis

To test the effects of different fertilization treatments on number of leaves, leaf herbivory, phytophagous mite density, and predatory mite occurrence (presence/absence) for each plant species, we used generalized linear mixed models (GLMMs). We used N and P levels as fixed categorical variables, also considering the interaction between the two nutrients. As some plant individuals were included after November 2018 (to compensate for high mortality, see above), the number of fertilization events that each plant received varied. Therefore, ‘number of fertilization events’ to which the plant was submitted was included as a covariate. To control for differences in plant size at the start of the experiment, in the model of plant number of leaves, we included initial height (i.e., height in the first fertilization event to which the plant was submitted) as covariate. When analyzing herbivory (leaf herbivory (%) and phytophagous mite density) and predation, to disentangle between effects of resource abundance from resource quality, we included ‘number of leaves’ as a covariate in the models. A significant effect of N or P over and above the effect of ‘number of leaves’ is then an indication of an effect driven by changes in quality of plant resources. As we had multiple measurements per plant individual, to account for the spatial and temporal structure of the data, we included “sampling date” and “plant identity nested within study site” as random effects. This structure controls for repeated measurements on the same individual over time, as well as potential site-level variation. For each metric, we also attempted to run a single GLMM for all species, with plant species and its interaction with P and N as terms in the model. Yet, our statistical power was not sufficient for such a large model.

For numbers of leaves, we assumed a negative binomial error structure (and log-link function). To analyze leaf herbivory (%) and abundance of phytophagous mites, we used Tweedie error structure (and log-link function). For phytophagous mites analyses, we considered only the individuals classified as phytophagous, but as a sensitivity test, we re-run the analyses also considering the mites with undetermined diet (e.g., juveniles, mites without full taxonomic ID from families with mixed feeding strategies). A binomial model was used to analyze the probability of occurrence of predatory mites. For each plant species, a posteriori analysis was performed to compare results under different combinations of P and N, using the "glht" function version 1.4–25 from the ‘multcomp’ package (Hothorn et al. 2008). All analyses were performed using “glmmTMB” v.1.1.4 (Brooks et al. 2017), in the R program version 4.2.1 (R Core Team 2023).

Whenever the probability of N or P having a significant effect in the models exceeded 90% (*P* value < 0.1), post hoc tests were performed to compare the values obtained in all pairwise combinations of nutrient levels. Values with a probability of being different greater than 95% (*P* < 0.05) are indicated with different letters in the figures.

## Results

### Effects of fertilization on plant vegetative metrics

**Fig. 1:**
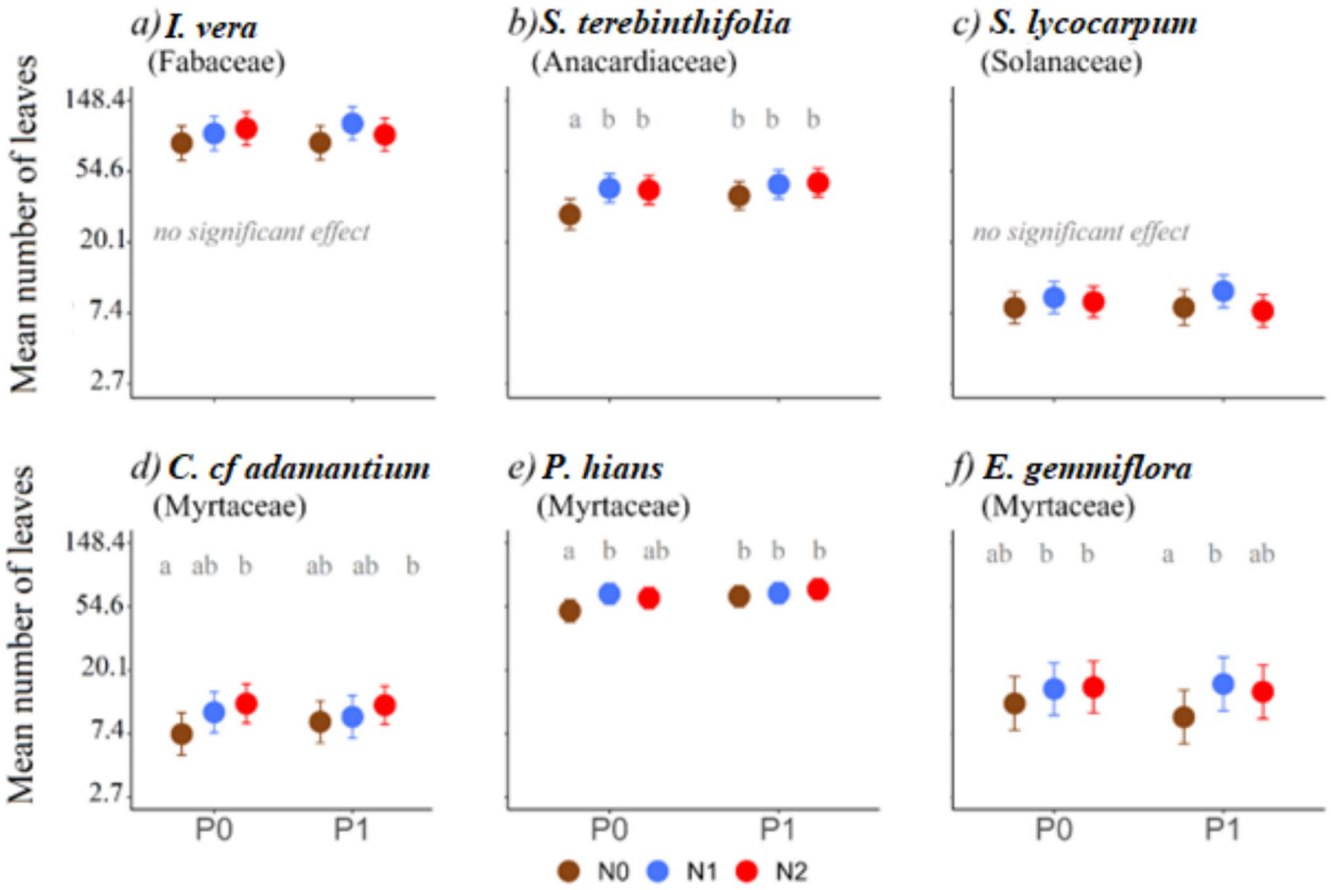
Effect of the different fertilization treatments on the number of leaves for each plant species. Mean estimates and associated 95% confidence intervals are shown. The *y*-axis presents back-transformed values: all estimates originally modeled on a logarithmic scale were converted back to their corresponding exponential units to facilitate interpretation. N0 = no N addition, N1 = 60 kg N/ha, N2 = 130 kg N/ha; P0 = no P addition, P1 = 40 kg P/ha. Whenever the probability of N or P having a significant effect exceeded 90% (*P* value < 0.1; Table S4), post hoc tests were done to compare values obtained under different nutrient combinations (values that have a probability of being different higher than 95%, i.e. *P* < 0.05, are indicated with distinct letters). Details of the statistical analyses are provided in Tables S4 and S5

For most species, N had a positive effect on the number of leaves, mean values being higher at the intermediate and/or highest N dosages (Fig. 1). Yet, some species were more affected than others (significant for *S. terebinthifolia*, *C. cf adamantium*, *P. hians* and *E. gemmiflora*, Fig. 1; Table S4), and no difference was detected between intermediate (N1) and highest (N2) levels of N (Fig. 1; Table S5). Also, for most species, no influence of P levels on the effect of N was detected (i.e., interaction term had no significant effect). Notably, for *S. terebinthifolia* and *P. hians*, increases in the number of leaves associated with P addition were observed only in the absence of N (i.e., under low N conditions; Fig. 1b, e). Under N-enriched conditions, P addition did not result in further increases in leaf number. Nevertheless, this interactive effect between N and P was weak as it was not detected when applying a penalization adjustment for multiple comparisons, nor supported by log-likelihood ratio tests of the full model (Tables S4 and S5).

The magnitude of treatment effects also differed across species. For *S. terebinthifolia*, the addition of N increased the number of leaves by more than 40% compared to the control, while the addition of P increased the number of leaves by 31%. Plants receiving both nutrients produced more than 50% leaves in comparison with those without any of these nutrients (control) (Fig. 1b; Table S4). For *C. cf adamantium*, the highest levels of N increased the number of leaves by approximately 60% in relation to control treatment (Fig. 1d; Table S4). For *P. hians*, N addition increased leaf number by 31% at N1 (Table S5). The addition of P increased leaf number by 26%, and in P-amended plants, N further increased leaf number by 32% (N1P1) and 41% (N2P1), even though the N × P interaction was not significant (Fig. 1e; Table S4).

For *E. gemmiflora*, N addition had a significant positive effect on mean leaf number, especially when P was added (Fig. 1f).

### Effects of fertilization on herbivores and higher trophic levels

**Fig. 2:**
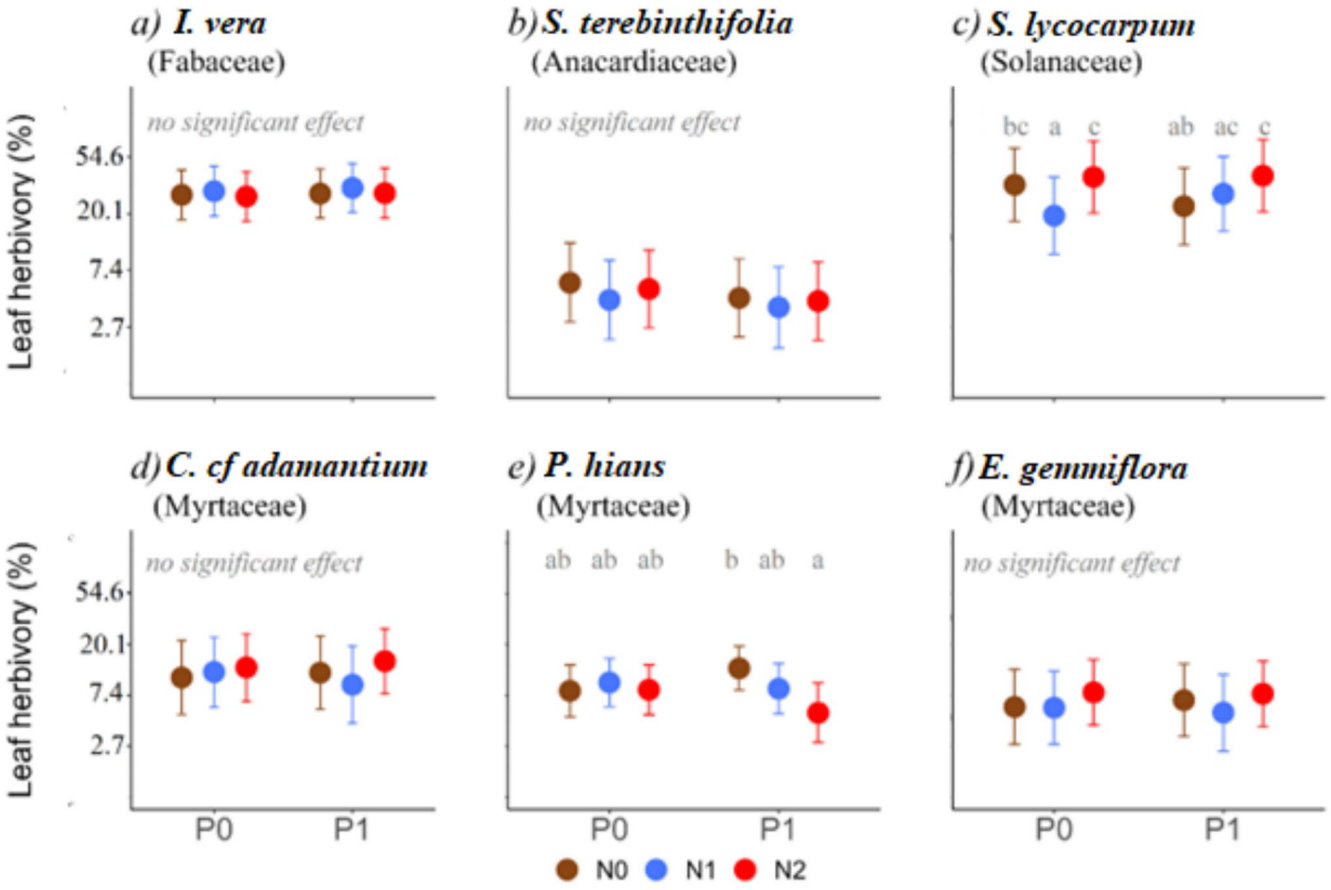
Effect of the different fertilization treatments in the percentage of leaves with herbivory marks, for each plant species. Mean estimates and associated 95% confidence intervals are shown. The *y*-axis presents back-transformed values: all estimates originally modeled on a logarithmic scale were converted back to their corresponding exponential units to facilitate interpretation. N0 = without addition of N, N1 = 60 kg N/ha, N2 = 130 kg N/ha, P0 = without addition P, P1 = 40 kg P/ha. Whenever the probability of N or P having a significant effect was higher than 90% (*P* value < 0.1, Table S4), post hoc tests were done to compare values obtained under different nutrient combinations (values that have a probability of being different higher than 95%, i.e. *P* < 0.05, are indicated with distinct letters). Details on statistical analyses are presented in Tables S4 and S5

Effects of fertilization treatments differed between herbivore types. For large foliar herbivores (mostly ants, caterpillars, and beetles), the *S. lycocarpum* showed the highest proportion of leaves with signs of herbivory (mean ± sd: 38 ± 36%), the addition of N led to a 42% reduction in herbivory in intermediate N level, while in the presence of P herbivory increased by 71% with highest N level (Fig. 2c). *I. vera* also had high levels of herbivory (31 ± 25% of the leaves affected), but no clear effect of fertilization treatment was detected (Fig. 2a; Table S4). The other species showed less than 15% of leaves with signs of herbivory [*C. cf adamantium* (13 ± 21%), *P. hians* (9 ± 8%), *E. gemmiflora* (8 ± 17%), *S. terebinthifolia* (6 ± 10%)]. Of these, only *P. hians* showed effects of fertilization; no effects were detected in the other species. Yet, contrary to what was detected for *S. lycocarpum*, the addition of N combined with P reduced herbivory regarding adding only P, this effect being significant when N was added in high dosages, reducing herbivory by 58%. (Fig. 2e).

**Fig. 3:**
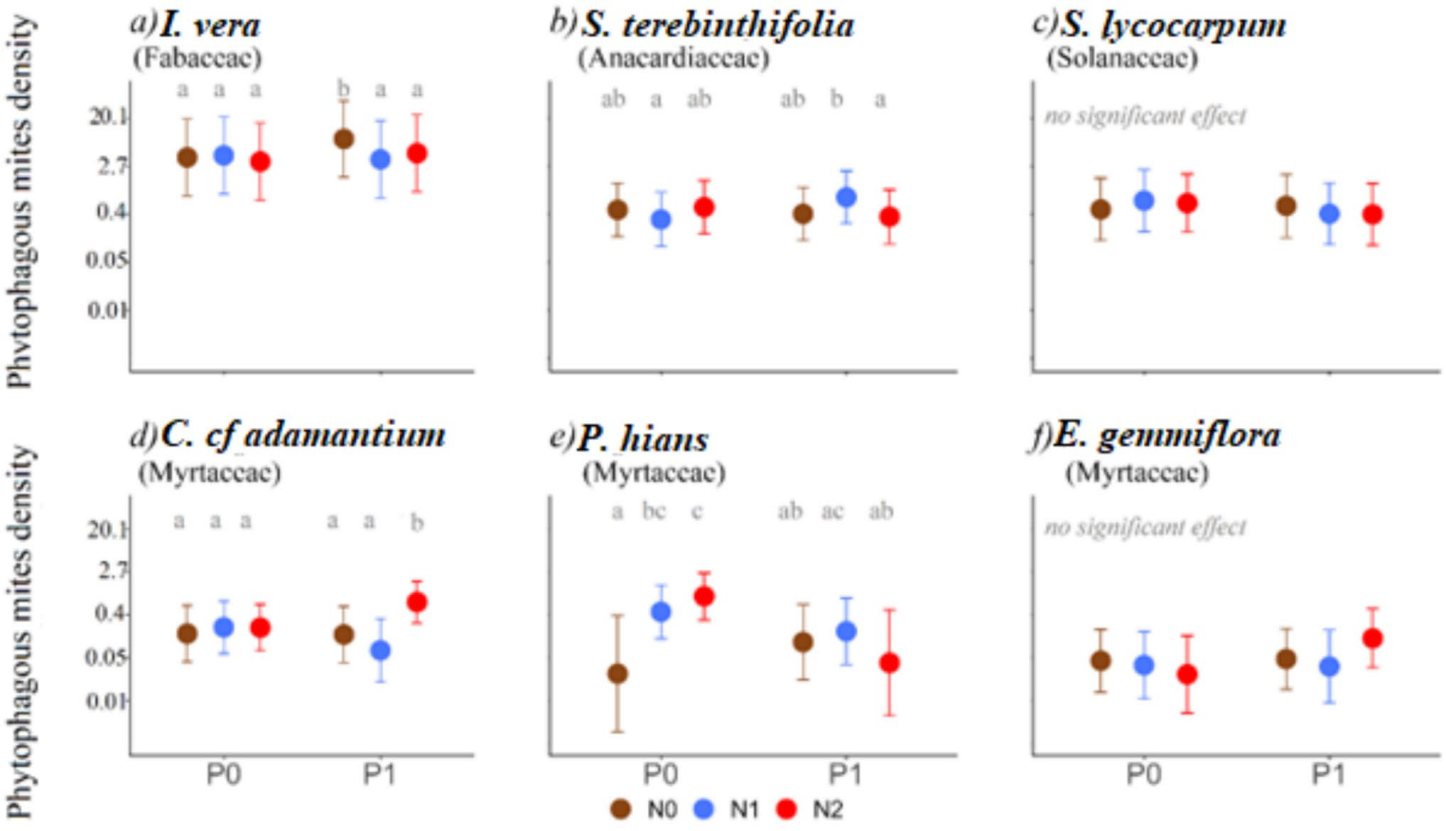
Effect of the fertilization treatments on phytophagous mite density (individuals per leaf), assuming mites with indeterminate feeding habits are also phytophagous, detected on each plant species. Mean estimates and associated 95% confidence intervals are shown. The *y*-axis presents back-transformed values: all estimates originally modeled on a logarithmic scale were converted back to their corresponding exponential units to facilitate interpretation. N0 = without addition of N, N1 = 60 kg N/ha, N2 = 130 kg N/ha, P0 = without addition P, P1 = 40 kg P/ha. Whenever the probability of N or P having a significant effect was higher than 90% (*P* value < 0.1, Table S4), post hoc tests were done to compare values obtained under different nutrient combinations (values that have a probability of being different higher than 95%, i.e. *P* < 0.05, are indicated with distinct letters). Details on statistical analyses are presented in Tables S4 and S5

As for mites, although their density was generally low (Table S6), effects of fertilizers were more frequently detected than in large invertebrate herbivores (Fig. 3, Table S4). *I. vera* was the species with the highest density of phytophagous mites (79% of all mites detected were found in this species), followed by *S. terebinthifolia* (12%), and the three species of Myrtaceae showing the lowest densities.

In total, we collected 11,976 mites belonging to 35 morphogroups in 16 families (Table S3). The phytophagous mites were the most abundant group, making up 86% of mites collected. Most (73%) mites belonged to the Tenuipalpidae family, followed by the Tetranychidae family (8%), both families of phytophagous mites, and by the predatory mite family Phytoseiidae (4%).

The effect of N input on the density of phytophagous mites depended on the levels of P, except for *S. lycocarpum* and *E. gemmiflora* for which no significant effects of N or P were detected*.* The combined addition of N and P increased the density of phytophagous mites compared to the addition of N alone by 152% (N1P1 vs. N1P0) for *S. terebinthifolia* and by 228% (N2P1 vs. N2P0) for *C. cf adamantium* (Fig. 3b; d, Table S5). For *P. hians*, in the absence of P, high levels of N increased mite density by 36 times, while this effect disappeared or became negative when P was introduced (Fig. 3e, Table S5). For *I. vera*, the addition of P increased mite density by more than 110%, but N input reduced this effect (Fig. 3a, Table S5). While for *S. lycocarpum*, no effect was detected when assuming that mites with indeterminate feeding habits are also phytophagous (Fig. 3c, Table S4), analyses focused only on mites with known feeding habits showed that N addition in the presence of P also had a negative effect to phytophagous mites density, with a reduction of more than 70% (Fig. S2e, Table S5). For the other plant species, there was consistency in the patterns observed for the density of phytophagous mites, regardless of whether indeterminate feeding mites were included or not (Figs. 3 and S2).

**Fig. 4:**
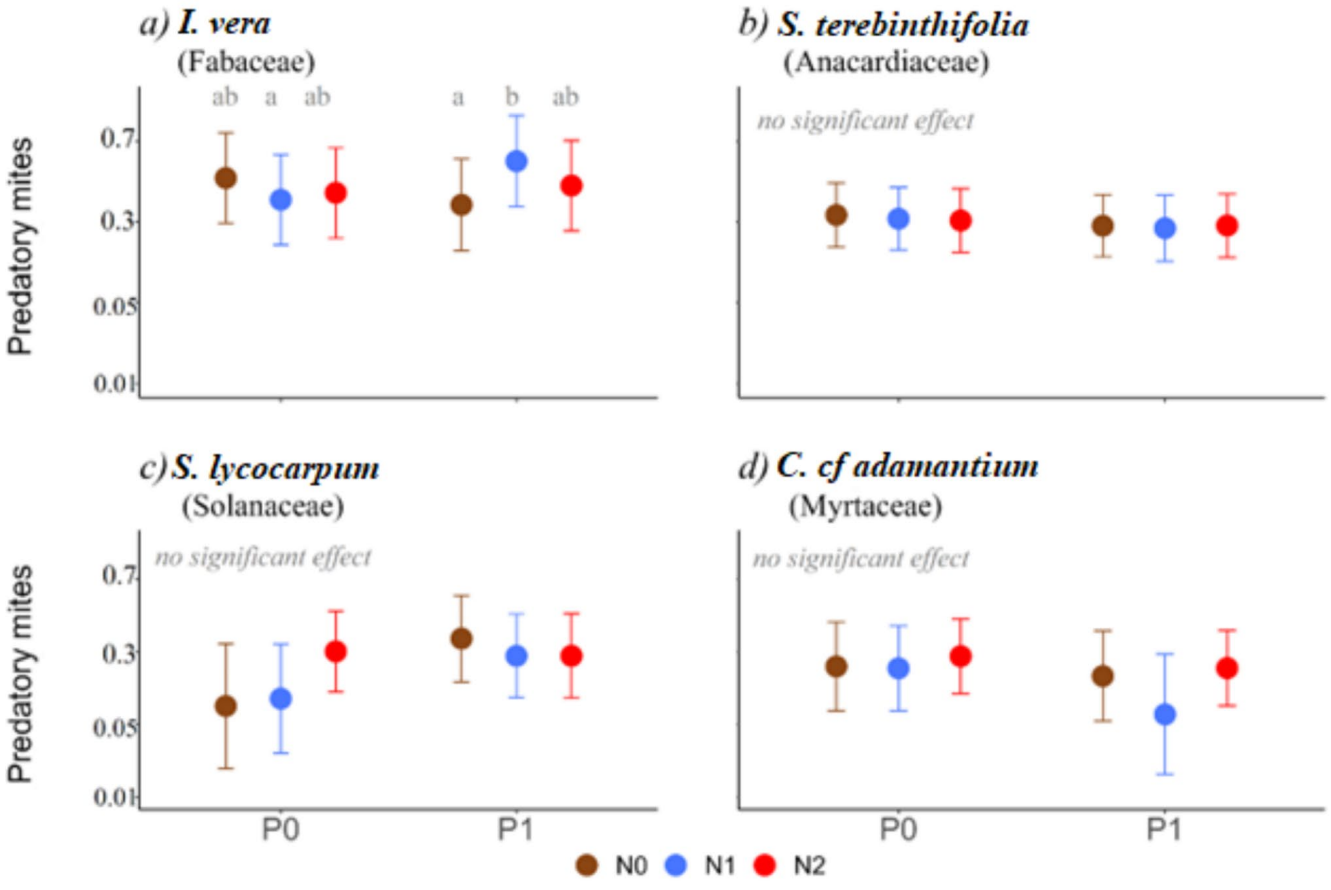
Effect of the different fertilization treatments in predatory mite probability of occurrence for each plant species. Mean estimates and associated 95% confidence intervals are shown. The y-axis presents back-transformed values: all estimates originally modeled on a logarithmic scale were converted back to their corresponding exponential units to facilitate interpretation. N0 = without addition of N, N1 = 60 kg N/ha, N2 = 130 kg N/ha, P0 = without addition P, P1 = 40 kg P/ha. The species *P. hians* and *E. gemmiflora* were not included in the analyses due to the low number of predatory mites detected. Whenever the probability of N or P having a significant effect was higher than 90% (*P* value < 0.1, Table S4), post hoc tests were done to compare values obtained under different nutrient combinations (values that have a probability of being different higher than 95%, i.e. *P* < 0.05, are indicated with distinct letters). Details on statistical analysis are presented in Tables S4 and S5

Most predatory mites were detected in *I. vera* (42% of the total number of predatory mites), *S. lycocarpum* (26%), *S. terebinthifolia* (19%), *C. cf adamantium* (9%), being less frequent in *E. gemmiflora* (2%) and *P. hians* (1%). As for the proportion of mites that were predatory (in relation to the total number of mites) *E. gemmiflora* (41% of their total number of mites), *S. lycocarpum* (39%) and *C. cf adamantium* (27%) were the plant species with higher values, predatory mites being less frequent in *P. hians* (12%), *S. terebinthifolia* (12%) and *I. vera* (4%). Due to the low number of mites in *P. hians* and *E. gemmiflora*, these two species were not used to analyze the effect of fertilization on predatory mites. Among the four remaining plant species, *I. vera* showed, in the presence of P and with the addition of an intermediate N level, an almost threefold increase in the occurrence of predatory mites (Fig. 4a; Table S5).

Overall, fertilization effects on leaf number, herbivory, and mite density were highly species-specific and did not follow a single directional pattern across trophic levels.

## Discussion

Our results show that soil nutrient enrichment does not generate a uniform response across Cerrado tree species, nor does it consistently propagate through herbivores to predatory mites. Instead, fertilization effects were strongly species-specific, with changes in plant vegetative metrics, herbivory, and phytophagous mite abundance occurring independently. This supports the idea that nutrient enrichment alters ecological interactions in a context-dependent manner, rather than following a single directional pathway across trophic levels.

### Effects of fertilization on plant vegetative metrics

While we detected an overall positive trend of N input in leaf count, the variations in response intensity among plant species indicate that, under a scenario of soil nutrient enrichment in Cerrado, certain native plant species may have a competitive advantage, and may increase their dominance, potentially driving others to local extinction. Such negative effects of nutrient addition are likely to affect more nitrophobous plants favoring nitrophilous plants (Bobbink et al. 2010). Identifying nitrophilous and nitrophobous plants in Cerrado can subsidize management plans and policies that aim to predict and minimize impacts on environmental eutrophication. Our results suggest that *C. cf. adamantium* and *P. hians*, two Myrtaceae species, exhibit a positive response to nitrogen addition, consistent with nitrophilous traits. Previous studies have shown that forest tree species within the Myrtaceae family respond positively to increased nitrogen availability, with enhanced survival (Nussbaumer et al. 2016) and biomass accumulation (Wooliver et al. 2016), indicating that high nitrogen responsiveness may be a common feature within the family. Although these two species occur naturally in biomes characterized by nutrient-poor soils, such as the Cerrado (Table S2), they have wide geographic distributions across Brazil, including regions, such as the Amazon and Atlantic Forest, where nutrient availability is enhanced by high organic matter inputs and efficient nutrient cycling (Table S2). This broad distribution across contrasting edaphic contexts supports the observed positive responses to nitrogen availability.

The results presented here may have implications for choices related to restoration programs. Plant responses to fertilization are closely related to their specific differences in nutritional requirement, such as co-limitation by different nutrients and differences in nutrient uptake and economy strategies, resulting in different intensities or even directions of responses (Kozovits et al. 2007; Wooliver et al. 2016). Yet, it is possible that such a beneficial effect only occurs when plants are protected from any competition effects with other species, as they were in our experimental setup. Further studies involving combinations of plants co-occurring and competing for soil resources would be needed to verify if such beneficial effect occurs in nature. Nevertheless, as *P. hians* is a fast-growing species and *C. cf adamantium* is a slow-growing species (Table S2), in natural conditions where plants compete for space and resources, we can assume that the first has a higher risk of increasing dominance over other native species under a scenario of soil nutrient enrichment. For the slow-growing Myrtaceae species which appear later in the ecological succession, *C. cf adamantium* and *E. gemmiflora* generally have a more conservative resource use strategy, being less able to respond to changes in nitrogen availability (Aidar et al. 2003; Báez and Homeier 2018). Therefore, *P. hians* is a species that may be better to avoid in restoration programs where soil is eutrophicated (e.g. due to nutrient leaching from farms).

The lack of a significant increase in the number of leaves in *I. vera*, despite increased soil nutrient levels, may reflect its ability to regulate nitrogen uptake through symbiotic N₂ fixation with rhizobia, maintaining nitrogen balance even in nutrient-poor soils (see Table S2). Previous research suggests that increased N fertilization can lead to a reduction in biological N fixation (e.g., Weber et al. 2007 and Xia et al. 2017), which may explain the limited vegetative response observed in this species under higher N availability.

Contrary to what we expected, we found no significant effect of P on the plant number of leaves of most plant species. Only *S. terebinthifolia* and *P. hians* showed a significant response to increases in N and P in a non-additive manner. Other studies did not find effects of P and its interaction with N, which may be linked to factors ranging from low availability for use in the soil (Bucci et al. 2006) to the presence of P conservation mechanisms for some plant species adapted to dystrophic environments (Kozovits et al. 2007; Abrahão et al. 2019). Yet, it is important to note that we found an effect of P on other trophic levels. Thus, P was likely allocated to functions other than biomass production, potentially enhancing leaf nutritional quality by increasing tissue P concentration without affecting leaf quantity. Indeed, nutrient enrichment can alter several tree characteristics in addition to aerial biomass, such as root biomass, concentrations of photosynthetic pigments, tissue nitrogen, concentrations of proteins carbohydrates, and secondary metabolites (Li et al. 2016), which in turn can affect interactions with higher trophic levels.

### Effects of fertilization on herbivores and higher trophic levels

Given the great variability of effects of fertilization on plant growth, variability on herbivore responses to fertilization was expected. While previous studies on tropical regions show that, increasing soil N availability (alone or in combination with P) has negative impacts on the abundance of terrestrial tropical invertebrates (Nessel et al. 2021, 2023), other studies in temperate ecosystems demonstrated that the effects of soil fertilization on herbivores can be positive, negative, or neutral (Kyto et al. 1996; Butler et al. 2012; La Pierre and Smith 2016; Nessel et al. 2021, 2023), depending on the host plant species and herbivorous group considered, as observed in our study. The species *I. vera* showed the highest density of mites and a high leaf herbivory regardless of the nutrient treatment, while the other species showed intermediate to high resistance to herbivory. This reflected in differences between levels of herbivory in response to soil nutrient enrichment for each plant studied, making it difficult to detect general response patterns.

As expected, the effects of nutrient supply were more pronounced for phytophagous mites than for plant growth. In general, we found a positive effect for plants. Other studies also point out that the effects can be magnified by trophic cascades (e.g., Carvalheiro et al. 2010).

The fact that the effects of nutrients were more accentuated for mites than for herbivory resulting from large invertebrate could be related to different levels of diet specialization. Sucking herbivores, such as phytophagous mites, showed a much stronger response to fertilizers than chewing insects, such as large external leaf herbivores (Butler et al. 2012). Sucking herbivores tend to be more specialized feeding on a single species or phylogenetically close plant species, while chewing herbivores can be more generalist (Ali and Agrawal 2012), and magnification of impacts through trophic chains or webs is mainly expected for species with more specialized diets (Carvalheiro et al. 2010). Differences in functional and behavioral traits (e.g., eating habit, mobility) could also influence the responses of herbivores to plant changes mediated by fertilizers (Kyto et al. 1996; Butler et al. 2012; Nessel et al. 2021).

As in other studies (Butler et al. 2012; Nessel et al. 2021; 2022), here it was difficult to detect a consistent effect of phosphorus and its interaction with N on herbivores and predators. Although the literature describes an overall negative effect (Butler et al. 2012; Nessel et al. 2021, 2023), some studies indicate positive effects of increasing P availability on herbivores (Butler et al. 2012). However, the effect of increasing P, especially in isolation, still needs to be further investigated.

The lack of a consistent increase in predatory mite presence, even in species showing higher herbivory or phytophagous mite density, suggests that top-down regulation plays a limited role under the fertilization scenarios tested here. This decoupling indicates that nutrient enrichment effects in this system are primarily driven by bottom-up processes acting directly on plants and herbivores, rather than by trophic cascades.

### Concluding remarks

Environmental eutrophication is one of the major human-driven changes affecting biodiversity (Bobbink et al. 2010; Steffen et al. 2015). Due to its intensive use for agriculture, Cerrado is highly exposed to such effect (Bustamante et al. 2012). Our results clearly show that altered soil conditions will favor some plant species more than others and will have consequences for higher trophic levels. These findings highlight the urge for long term monitoring schemes to evaluate how ongoing soil nutrient enrichment is changing the dynamics between plant trees that naturally occur in Cerrado and their ecological interactions. These findings also have implications for restoration programs in Cerrado. Soil properties in areas that are subjected to restoration actions typically have highly degraded soils (Prescott et al. 2021), potentially being poorer in nutrients than normal soils or potentially being enriched in certain nutrients that are widely applied in crop fields (nitrogen and phosphorus). Future studies with a larger number of species that explore which plant traits explain their response to nutrient level changes are essential to identify how different plant species will perform when used in a restoration plan. Overall, the results of this work contribute to a better understanding of how different plant species, valuable for restoration, respond when planted in soils with different nutrient conditions.

## Supplementary Information

Below is the link to the electronic supplementary material.

Supplementary file1 (DOCX 1417 KB)

## Data Availability

The datasets used and/or analyzed during the current study are available from the corresponding author on reasonable request.
