# Supplementary material for: Effects of soil nutrient enrichment on biomass, herbivores, and their predators differ between tree species in the Brazilian Cerrado
Source: Oecologia. 2026 Feb 7;208(2):33. doi: 10.1007/s00442-026-05863-z (PMC12880998; doi:10.1007/s00442-026-05863-z)
Supplement: Supplementary file 1 — Supplementary file1 (DOCX 1417 KB) [file 442_2026_5863_MOESM1_ESM.docx]

**Supporting Information**

**Table S1.** Geographic coordinates (decimal degrees) of the five study sites where each replica of the experiment is located.

| **Study sites** | **Longitude** | **Latitude** |
| --- | --- | --- |
| Greenhouse of the Ecology Department UFG | -49.258889 | -16.603056 |
| UFG Veterinary and zootechnics school | -49.275556 | -16.598056 |
| Horticulture of the UFG School of Agronomy | -49.281389 | -16.595833 |
| UFG forestry engineering school nursery | -49.290556 | -16.592778 |
| UFG agroecological development center | -49.286389 | -16.599167 |

**Table S2**. Details of plant species used in the experiment and their ecological characteristics. Species were classified in terms of their successional group (pioneer, P, early secondary, ES, late secondary, LS, secondary, S); phenological group (semideciduous, SD, deciduous, D, evergreen, E); N fixing ability, life form, height, geographical domains (Amazon, AM, Caatinga, CA, Cerrado, CE, Atlantic Forest, AF, Pampa, PAM, Pantanal, PAN); habitat =(forest, F, savanna, S, grassland, G), growth speed. Referene sources used to gather the information are indicated.

| **Species** | **Family** | **Popular name** | **Successional group** | **Phenological group** | **N fixing** | **life form** | **Height (m)** | **Domains** | **habitat** | **growth** | **Source** |
| --- | --- | --- | --- | --- | --- | --- | --- | --- | --- | --- | --- |
| *Psidium hians* Mart. ex DC.. | Myrtaceae | araçá | ES | SD | No | shurb-tree | 1.0-3.0 | AM, CE | F/S | fast | 1,2,3,4,5, 8 |
| *Eugenia gemmiflora* O.Berg | Myrtaceae | Cagaita-vermelha | LS | E | No | sub/ shurb-tree | 0.1-15 | CA, CE | S | slow | 2,3,5,6,13, 14, 8 |
| *Campomanesia cf adamantium* (Cambess.) O.Berg | Myrtaceae | gabiroba | LS | D | No | Sub/shurb | 0.3-2.0 | CE, AF | S/G | slow | 2,5,13,8 |
| *Schinus terebinthifolia* Raddi | Anacardiaceae | aroeira-vermelha/ pimenteira | P | E | No | shurb-tree | 5.0-10.0 | CA, CE, AF, PAM | F | very fast | 1,2,3,5,6,9 |
| *Inga vera subsp. affinis (DC.) T.D.Penn.* | Fabaceae | ingá | P/ ES | SD | Yes (rhizobia) | tree | 5.0-25.0 | AM, CE, AF; PAN | F | very fast | 3,5,6,7 |
| *Solanum lycocarpum* A.St.-Hil. | Solanaceae | lobeira | P | E/ D | No | shurb-tree | 1.0-5.0 | CE, AF | S/G | fast | 2,3,5,6,10-12 |
| Source = 1. Guedes and Kruped, 2017; 2. Kuhlmann, 2018, 3. de Almeida and Viani, 2021; 4. Bezerra et al., 2018; 5. https://floradobrasil.jbrj.gov.br; 6. Lorenzi, 2016; 7. Carvalho,2008; 8. Personal observation (increase in height during the experiment); 9. Carvalho, 2003; 10. Carvalho, 2010; 11. Carvalho,2010; 12. Oliveira et al., 2004 ; 13. Almeida, 2022; 14. Isernhagen, 2015. | | | | | | | | | | | |

**Table S3.** List of mite morphospecies detected during this study. Information on mite family, morphogroup, feeding guild (phy= phytophagous, pre= predatory, und= undetermined/unknown) and abundance detected by plant species is provided.

| Family | Morphogroups | Feeding guilds | *I. vera* (Fabaceae) | *S. terebinthifolia* (Anacardiaceae) | *S. lycocarpum* (Solanaceae) | *C. cf adamantium* (Myrtaceae) | *P. hians* (Myrtaceae) | *E. gemmiflora* (Myrtaceae) | **Total** |
| --- | --- | --- | --- | --- | --- | --- | --- | --- | --- |
| Eriophyidae | spp. | Phy | 403 | 34 | 118 | 0 | 0 | 0 | **555** |
| Tenuipalpidae | *Brevipalpus* sp. | Phy | 2617 | 384 | 27 | 14 | 26 | 2 | **3070** |
| Tenuipalpidae | *Raoiella* spp. | Phy | 2 | 1 | 0 | 0 | 0 | 0 | **3** |
| Tenuipalpidae | Tenuipalpidae spp. | Phy | 4823 | 729 | 59 | 24 | 28 | 3 | **5666** |
| Tenuipalpidae | *Tenuipalpus* sp. | Phy | 20 | 1 | 0 | 0 | 0 | 0 | **21** |
| Tetranychidae | *Allonychus* spp. | Phy | 1 | 0 | 5 | 6 | 1 | 1 | **14** |
| Tetranychidae | *Atrichoproctus* spp. | Phy | 4 | 0 | 0 | 0 | 0 | 0 | **4** |
| Tetranychidae | *Eutetranychus* spp. | Phy | 0 | 1 | 1 | 0 | 0 | 0 | **2** |
| Tetranychidae | *Oligonychus* spp. | Phy | 5 | 0 | 2 | 2 | 0 | 0 | **9** |
| Tetranychidae | *Panonychus* spp. | Phy | 1 | 0 | 0 | 0 | 0 | 0 | **1** |
| Tetranychidae | Tetranychidae spp. | Phy | 743 | 36 | 67 | 76 | 3 | 3 | **928** |
| Tydeidae | *Lorryia formosa* | Phy | 0 | 1 | 0 | 0 | 0 | 0 | **1** |
| Tydeidae | *Lorryia* spp. | Phy | 5 | 2 | 1 | 1 | 0 | 0 | **9** |
| Ascidae | Ascidae spp. | Pre | 1 | 0 | 2 | 1 | 0 | 2 | **6** |
| Cheyletidae | Cheyletidae spp. | Pre | 4 | 1 | 0 | 1 | 0 | 0 | **6** |
| Cunaxidae | Cunaxidae spp. | Pre | 0 | 0 | 0 | 0 | 1 | 0 | **1** |
| Iolinidae | Iolinidae spp. | Pre | 93 | 72 | 203 | 22 | 4 | 1 | **395** |
| Mesostigmata | Mesostigmata spp. | Pre | 1 | 0 | 0 | 0 | 0 | 0 | **1** |
| Phytoseiidae | *Amblydromalus* spp. | Pre | 1 | 1 | 0 | 0 | 0 | 0 | **2** |
| Phytoseiidae | *Amblyseius* spp. | Pre | 12 | 2 | 0 | 0 | 0 | 0 | **14** |
| Phytoseiidae | *Euseius* spp. | Pre | 9 | 0 | 0 | 3 | 0 | 1 | **13** |
| Phytoseiidae | *Iphiseiodes* spp. | Pre | 26 | 7 | 1 | 5 | 0 | 1 | **40** |
| Phytoseiidae | *Phytoseius* spp. | Pre | 4 | 0 | 0 | 0 | 0 | 0 | **4** |
| Phytoseiidae | *Silvaseius* spp. | Pre | 1 | 0 | 0 | 0 | 0 | 0 | **1** |
| Phytoseiidae | Phytoseiidae sp1 | Pre | 0 | 1 | 0 | 0 | 0 | 0 | **1** |
| Phytoseiidae | Phytoseiidae sp2 | Pre | 1 | 0 | 0 | 0 | 0 | 0 | **1** |
| Phytoseiidae | Phytoseiidae spp. | Pre | 204 | 86 | 29 | 48 | 3 | 11 | **381** |
| Phytoseiidae | *Typhlodromalus* spp. | Pre | 0 | 0 | 0 | 2 | 0 | 0 | **2** |
| Stigmaeidae | Stigmaeidae spp. | Pre | 19 | 0 | 0 | 0 | 0 | 0 | **19** |
| Acaridae | Acaridae spp. | Und | 75 | 0 | 3 | 2 | 0 | 1 | **81** |
| Astigmata | Astigmata spp. | Und | 32 | 2 | 1 | 9 | 0 | 0 | **44** |
| Tarsonemidae | Tarsonemidae spp. | Und | 4 | 8 | 16 | 3 | 0 | 1 | **32** |
| Tydeidae | Tydeidae spp. | Und | 31 | 20 | 6 | 25 | 1 | 2 | **85** |
| NI | NI | Und | 347 | 66 | 46 | 50 | 1 | 10 | **520** |
| Oribatida | Oribatida spp. | Und | 10 | 5 | 7 | 2 | 0 | 0 | **24** |
| Winterschimidtiidae | Winterschimidtiidae spp. | Und | 14 | 0 | 3 | 3 | 0 | 0 | **20** |
| **Total** |  |  | **9513** | **1460** | **597** | **299** | **68** | **39** | **11976** |

**Table S4.** Statistical details for models evaluating the effects of fertilization on plant leaf number, herbivory, and predation for the six Cerrado plant species. (:) indicates an interaction term between two explanatory variables. P values are presented for each term of the model, extracted from log-likelihood ratio tests. NA= variable not included in the model. df.residual= Residual degrees-of-freedom.

| **Response variable** | **Species** | **N** | **P** | **N:P** | **Log (Leaves number)** | **Height (cm)** | **Number of fertilizations** | **df.residual** |
| --- | --- | --- | --- | --- | --- | --- | --- | --- |
| **Number of leaves** | | | | | | | |  |
|  | *I. vera* | 0.1215 | 0.8033 | 0.5368 | NA | 0.0082 | 0.0003 | 250 |
|  | *S. terebinthifolia* | **0.0001** | **0.0277** | 0.3188 | NA | <0.0001 | <0.0001 | 257 |
|  | *S. lycocarpum* | 0.1847 | 0.9123 | 0.5547 | NA | <0.0001 | <0.0001 | 185 |
|  | *C. cf adamantium* | **0.0128** | 0.8076 | 0.5674 | NA | <0.0001 | 0.6519 | 222 |
|  | *P. hians* | **0.0356** | **0.0287** | 0.2842 | NA | 0.2359 | NA | 51 |
|  | *E. gemmiflora* | **0.0192** | 0.5262 | 0.6934 | NA | 0.0002 | NA** | 209 |
| **Leaf herbivory (%)** | | | | | | | |  |
|  | *I. vera* | 0.5314 | 0.5431 | 0.9753 | 0.0028 | NA | 0.1857 | 244 |
|  | *S. terebinthifolia* | 0.5432 | 0.2177 | 0.9465 | 0.0009 | NA | 0.0004 | 258 |
|  | *S. lycocarpum* | **0.0050** | 0.9655 | **0.064** | <0.0001 | NA | 0.2819 | 184 |
|  | *C. cf adamantium* | 0.6708 | 0.8745 | 0.8469 | 0.3868 | NA | <0.0001 | 214 |
|  | *P. hians* | 0.1347 | 0.9931 | **0.0941** | 0.7237 | NA | NA | 50 |
|  | *E. gemmiflora* | 0.6186 | 0.9404 | 0.9258 | 0.0162 | NA | 0.0612 | 201 |
| **Phytophagous mites density** | | | | | | | |  |
|  | *I. vera* (with outlier) | 0.2232 | **0.0168** | 0.5762 | 0.4876 | NA | 0.0011 | 248 |
|  | *I. vera* (without outlier) | **0.0725** | **0.0433** | 0.1439 | 0.2763 | NA | 0.0081 | 249 |
|  | *S. terebinthifolia* | 0.7835 | 0.6010 | **0.0241** | 0.3946 | NA | 0.0089 | 262 |
|  | *S. lycocarpum* (with outlier) | 0.2903 | 0.1232 | **0.0484** | 0.0697 | NA | 0.3009 | 164 |
|  | *S. lycocarpum* (without outlier) | 0.5890 | 0.2490 | **0.0625** | 0.1781 | NA | 0.2850 | 163 |
|  | *C. cf adamantium* | **0.0483** | 0.3337 | 0.2468 | 0.1371 | NA | 0.5886 | 191 |
|  | *P. hians* | 0.3341 | **0.0819** | **0.0900** | 0.4385 | NA | NA | 50 |
| **Phytophagous mites density (assuming mites with indeterminate feeding habits are also phytophagous)** | | | | | | | |  |
|  | *I. vera* (with outlier) | 0.1754 | **0.0184** | 0.3548 | 0.5689 | NA | 0.0001< | 247 |
|  | *I. vera* (without outlier) | **0.0647** | **0.0358** | **0.0620** | 0.2991 | NA | 0.0010 | 246 |
|  | *S. terebinthifolia* | 0.7157 | 0.5374 | **0.0321** | 0.5575 | NA | 0.0022 | 262 |
|  | *S. lycocarpum* (with outlier) | 0.7647 | 0.1113 | 0.4379 | 0.0046 | NA | 0.3979 | 163 |
|  | *S. lycocarpum* (without outlier) | 0.9877 | 0.2665 | 0.5962 | 0.0182 | NA | 0.3799 | 162 |
|  | *C. cf adamantium* (with outlier) | **0.0632** | 0.9854 | 0.1060 | 0.2481 | NA | 0.7257 | 187 |
|  | *C. cf adamantium* (without outlier) | **0.0227** | 0.2825 | **0.0477** | 0.3758 | NA | 0.2997 | 190 |
|  | *P. hians* | 0.2196 | 0.1279 | **0.0669** | 0.4282 | NA | NA | 50 |
|  | *E. gemmiflora* | 0.5432 | 0.3162 | 0.3693 | 0.0168 | NA | 0.1244 | 193 |
|  |  |  |  |  |  |  |  |  |
| **Predators mites occurrence** | | | | | | | |  |
|  | *I. vera* | 0.7494 | 0.5751 | **0.0534** | 0.0018 | NA | 0.1440 | 252 |
|  | *S. terebinthifolia* | 0.9750 | 0.4635 | 0.9772 | 0.0002 | NA | 0.6883 | 264 |
|  | *S. lycocarpum* | 0.4629 | 0.1072 | 0.1415 | 0.8733 | NA | 0.4663 | 166 |
|  | *C. cf adamantium* | 0.4258 | 0.1953 | 0.5982 | 0.5020 | NA | 0.9999 | 193 |

**Variable not included as fixed in the model for better model fit

**Table S5.** Results of post-hoc test between different treatments. Results were obtained using glht function from multcomp R package (Hothorn et al., 2016).P-values are presented with (adj) and without the adjustment for the number of comparisons.

|  | **contrast** | **Estimate** | **Std. Error** | **z value** | **Pr(>\|z\|) adj** | **Pr(>\|z\|)** |
| --- | --- | --- | --- | --- | --- | --- |
| **Number of leaves** |  |  |  |  |  |  |
| *S. terebinthifolia* | N1.P0 - N0.P0 == 0 | 0.37155 | 0.10373 | 3.582 | **0.00461** | **0.000341** |
|  | N2.P0 - N0.P0 == 0 | 0.34687 | 0.10308 | 3.365 | **0.00994** | **0.000765** |
|  | N0.P1 - N0.P0 == 0 | 0.26658 | 0.10807 | 2.467 | 0.13355 | **0.013633** |
|  | N1.P1 - N0.P0 == 0 | 0.42590 | 0.11016 | 3.866 | **0.00152** | **0.000111** |
|  | N2.P1 - N0.P0 == 0 | 0.44950 | 0.10503 | 4.280 | **< 0.001** | **< 0.0001** |
|  | N2.P0 - N1.P0 == 0 | -0.02468 | 0.09950 | -0.248 | 0.99987 | 0.804078 |
|  | N0.P1 - N1.P0 == 0 | -0.10497 | 0.09850 | -1.066 | 0.89470 | 0.286550 |
|  | N1.P1 - N1.P0 == 0 | 0.05434 | 0.10352 | 0.525 | 0.99518 | 0.599614 |
|  | N2.P1 - N1.P0 == 0 | 0.07795 | 0.09936 | 0.784 | 0.97012 | 0.432766 |
|  | N0.P1 - N2.P0 == 0 | -0.08029 | 0.09924 | -0.809 | 0.96585 | 0.418485 |
|  | N1.P1 - N2.P0 == 0 | 0.07903 | 0.10376 | 0.762 | 0.97373 | 0.446276 |
|  | N2.P1 - N2.P0 == 0 | 0.10263 | 0.09966 | 1.030 | 0.90777 | 0.303099 |
|  | N1.P1 - N0.P1 == 0 | 0.15932 | 0.10109 | 1.576 | 0.61386 | 0.115021 |
|  | N2.P1 - N0.P1 == 0 | 0.18292 | 0.09724 | 1.881 | 0.41313 | **0.059940** |
|  | N2.P1 - N1.P1 == 0 | 0.02360 | 0.10072 | 0.234 | 0.99990 | 0.814725 |
|  |  |  |  |  |  |  |
| *C. cf adamantium* | N1.P0 - N0.P0 == 0 | 0.34183 | 0.18234 | 1.875 | 0.4171 | **0.06083** |
|  | N2.P0 - N0.P0 == 0 | 0.47792 | 0.17863 | 2.675 | **0.0799** | **0.00746** |
|  | N0.P1 - N0.P0 == 0 | 0.19128 | 0.18896 | 1.012 | 0.9138 | 0.31142 |
|  | N1.P1 - N0.P0 == 0 | 0.27105 | 0.18887 | 1.435 | 0.7048 | 0.15124 |
|  | N2.P1 - N0.P0 == 0 | 0.45545 | 0.17482 | 2.605 | **0.0954** | **0.00918** |
|  | N2.P0 - N1.P0 == 0 | 0.13609 | 0.17179 | 0.792 | 0.9688 | 0.42826 |
|  | N0.P1 - N1.P0 == 0 | -0.15055 | 0.18352 | -0.820 | 0.9638 | 0.41204 |
|  | N1.P1 - N1.P0 == 0 | -0.07077 | 0.18230 | -0.388 | 0.9989 | 0.69784 |
|  | N2.P1 - N1.P0 == 0 | 0.11362 | 0.16930 | 0.671 | 0.9850 | 0.50215 |
|  | N0.P1 - N2.P0 == 0 | -0.28664 | 0.17966 | -1.595 | 0.6008 | 0.11061 |
|  | N1.P1 - N2.P0 == 0 | -0.20687 | 0.17972 | -1.151 | 0.8594 | 0.24972 |
|  | N2.P1 - N2.P0 == 0 | -0.02247 | 0.16385 | -0.137 | 1.0000 | 0.89090 |
|  | N1.P1 - N0.P1 == 0 | 0.07977 | 0.19072 | 0.418 | 0.9984 | 0.67574 |
|  | N2.P1 - N0.P1 == 0 | 0.26417 | 0.17514 | 1.508 | 0.6582 | 0.13148 |
|  | N2.P1 - N1.P1 == 0 | 0.18439 | 0.17752 | 1.039 | 0.9046 | 0.29895 |
|  |  |  |  |  |  |  |
| *P. hians* | N1.P0 - N0.P0 == 0 | 0.26981 | 0.10049 | 2.685 | **0.0781** | **0.007255** |
|  | N2.P0 - N0.P0 == 0 | 0.20300 | 0.10523 | 1.929 | 0.3835 | **0.053723** |
|  | N0.P1 - N0.P0 == 0 | 0.23098 | 0.10565 | 2.186 | 0.2435 | **0.028788** |
|  | N1.P1 - N0.P0 == 0 | 0.28121 | 0.10283 | 2.735 | **0.0684** | **0.006242** |
|  | N2.P1 - N0.P0 == 0 | 0.34164 | 0.09864 | 3.464 | **0.0071** | **0.000533** |
|  | N2.P0 - N1.P0 == 0 | -0.06681 | 0.09767 | -0.684 | 0.9837 | 0.493963 |
|  | N0.P1 - N1.P0 == 0 | -0.03883 | 0.09779 | -0.397 | 0.9987 | 0.691353 |
|  | N1.P1 - N1.P0 == 0 | 0.01141 | 0.09635 | 0.118 | 1.0000 | 0.905744 |
|  | N2.P1 - N1.P0 == 0 | 0.07184 | 0.09546 | 0.752 | 0.9751 | 0.451751 |
|  | N0.P1 - N2.P0 == 0 | 0.02798 | 0.09711 | 0.288 | 0.9997 | 0.773247 |
|  | N1.P1 - N2.P0 == 0 | 0.07821 | 0.09670 | 0.809 | 0.9659 | 0.418618 |
|  | N2.P1 - N2.P0 == 0 | 0.13864 | 0.09846 | 1.408 | 0.7216 | 0.159085 |
|  | N1.P1 - N0.P1 == 0 | 0.05023 | 0.09668 | 0.520 | 0.9954 | 0.603358 |
|  | N2.P1 - N0.P1 == 0 | 0.11066 | 0.09864 | 1.122 | 0.8722 | 0.261928 |
|  | N2.P1 - N1.P1 == 0 | 0.06043 | 0.09674 | 0.625 | 0.9892 | 0.532212 |
|  |  |  |  |  |  |  |
| *E. gemmiflora* | N1.P0 - N0.P0 == 0 | 0.233714 | 0.191155 | 1.223 | 0.826 | 0.2215 |
|  | N2.P0 - N0.P0 == 0 | 0.278119 | 0.186939 | 1.488 | 0.672 | 0.1368 |
|  | N0.P1 - N0.P0 == 0 | -0.177313 | 0.195452 | -0.907 | 0.945 | 0.3643 |
|  | N1.P1 - N0.P0 == 0 | 0.287553 | 0.196090 | 1.466 | 0.685 | 0.1425 |
|  | N2.P1 - N0.P0 == 0 | 0.189726 | 0.192064 | 0.988 | 0.922 | 0.3232 |
|  | N2.P0 - N1.P0 == 0 | 0.044405 | 0.181851 | 0.244 | 1.000 | 0.8071 |
|  | N0.P1 - N1.P0 == 0 | -0.411027 | 0.191200 | -2.150 | 0.261 | **0.0316** |
|  | N1.P1 - N1.P0 == 0 | 0.053839 | 0.190481 | 0.283 | 1.000 | 0.7774 |
|  | N2.P1 - N1.P0 == 0 | -0.043988 | 0.188283 | -0.234 | 1.000 | 0.8153 |
|  | N0.P1 - N2.P0 == 0 | -0.455432 | 0.187457 | -2.430 | 0.146 | **0.0151** |
|  | N1.P1 - N2.P0 == 0 | 0.009433 | 0.186186 | 0.051 | 1.000 | 0.9596 |
|  | N2.P1 - N2.P0 == 0 | -0.088394 | 0.183488 | -0.482 | 0.997 | 0.6300 |
|  | N1.P1 - N0.P1 == 0 | 0.464865 | 0.196747 | 2.363 | 0.169 | **0.0181** |
|  | N2.P1 - N0.P1 == 0 | 0.367038 | 0.192526 | 1.906 | 0.398 | **0.0566** |
|  | N2.P1 - N1.P1 == 0 | -0.097827 | 0.191981 | -0.510 | 0.996 | 0.6104 |
| **Leaf herbivory (%)** |  |  |  |  |  |  |
| *S. lycocarpum* | N1.P0 - N0.P0 == 0 | -0.54627 | 0.22641 | -2.413 | 0.1498 | **0.01583** |
|  | N2.P0 - N0.P0 == 0 | 0.13710 | 0.18851 | 0.727 | 0.9783 | 0.46706 |
|  | N0.P1 - N0.P0 == 0 | -0.37897 | 0.22108 | -1.714 | 0.5190 | **0.08650** |
|  | N1.P1 - N0.P0 == 0 | -0.15966 | 0.20956 | -0.762 | 0.9734 | 0.44613 |
|  | N2.P1 - N0.P0 == 0 | 0.15835 | 0.19189 | 0.825 | 0.9624 | 0.40924 |
|  | N2.P0 - N1.P0 == 0 | 0.68337 | 0.22156 | 3.084 | **0.0246** | **0.00204** |
|  | N0.P1 - N1.P0 == 0 | 0.16730 | 0.24961 | 0.670 | 0.9850 | 0.50269 |
|  | N1.P1 - N1.P0 == 0 | 0.38661 | 0.23832 | 1.622 | 0.5805 | 0.10475 |
|  | N2.P1 - N1.P0 == 0 | 0.70463 | 0.22655 | 3.110 | **0.0226** | **0.00187** |
|  | N0.P1 - N2.P0 == 0 | -0.51607 | 0.21495 | -2.401 | 0.1539 | **0.01635** |
|  | N1.P1 - N2.P0 == 0 | -0.29675 | 0.20142 | -1.473 | 0.6785 | 0.14066 |
|  | N2.P1 - N2.P0 == 0 | 0.02126 | 0.18383 | 0.116 | 1.0000 | 0.90795 |
|  | N1.P1 - N0.P1 == 0 | 0.21931 | 0.23776 | 0.922 | 0.9401 | 0.35631 |
|  | N2.P1 - N0.P1 == 0 | 0.53732 | 0.21804 | 2.464 | 0.1332 | **0.01372** |
|  | N2.P1 - N1.P1 == 0 | 0.31801 | 0.20604 | 1.543 | 0.6329 | 0.12272 |
|  |  |  |  |  |  |  |
| *P. hians* | N1.P0 - N0.P0 == 0 | 0.16526 | 0.30418 | 0.543 | 0.9943 | 0.58692 |
|  | N2.P0 - N0.P0 == 0 | 0.02178 | 0.30500 | 0.071 | 1.0000 | 0.94307 |
|  | N0.P1 - N0.P0 == 0 | 0.44423 | 0.28038 | 1.584 | 0.6053 | 0.11310 |
|  | N1.P1 - N0.P0 == 0 | 0.04241 | 0.31176 | 0.136 | 1.0000 | 0.89179 |
|  | N2.P1 - N0.P0 == 0 | -0.43267 | 0.35569 | -1.216 | 0.8268 | 0.22382 |
|  | N2.P0 - N1.P0 == 0 | -0.14348 | 0.28519 | -0.503 | 0.9960 | 0.61489 |
|  | N0.P1 - N1.P0 == 0 | 0.27897 | 0.25834 | 1.080 | 0.8880 | 0.28020 |
|  | N1.P1 - N1.P0 == 0 | -0.12285 | 0.28099 | -0.437 | 0.9979 | 0.66196 |
|  | N2.P1 - N1.P0 == 0 | -0.59793 | 0.32185 | -1.858 | 0.4249 | **0.06320** |
|  | N0.P1 - N2.P0 == 0 | 0.42245 | 0.26789 | 1.577 | 0.6103 | 0.11480 |
|  | N1.P1 - N2.P0 == 0 | 0.02063 | 0.29329 | 0.070 | 1.0000 | 0.94392 |
|  | N2.P1 - N2.P0 == 0 | -0.45445 | 0.33491 | -1.357 | 0.7500 | 0.17480 |
|  | N1.P1 - N0.P1 == 0 | -0.40182 | 0.26726 | -1.503 | 0.6587 | 0.13271 |
|  | N2.P1 - N0.P1 == 0 | -0.87690 | 0.31228 | -2.808 | **0.0552** | **0.00498** |
|  | N2.P1 - N1.P1 == 0 | -0.47508 | 0.32909 | -1.444 | 0.6972 | 0.14885 |
| **Phytophagous mites density** |  |  |  |  |  |  |
| *I. vera* (with outlier) | N1.P0 - N0.P0 == 0 | -0.060543 | 0.346883 | -0.175 | 1.0000 | 0.86145 |
|  | N2.P0 - N0.P0 == 0 | -0.211274 | 0.350132 | -0.603 | 0.9908 | 0.54624 |
|  | N0.P1 - N0.P0 == 0 | 0.740510 | 0.335887 | 2.205 | 0.2354 | **0.02748** |
|  | N1.P1 - N0.P0 == 0 | 0.186231 | 0.343021 | 0.543 | 0.9944 | 0.58719 |
|  | N2.P1 - N0.P0 == 0 | 0.193970 | 0.340610 | 0.569 | 0.9930 | 0.56903 |
|  | N2.P0 - N1.P0 == 0 | -0.150731 | 0.348617 | -0.432 | 0.9981 | 0.66547 |
|  | N0.P1 - N1.P0 == 0 | 0.801053 | 0.334378 | 2.396 | 0.1576 | **0.01659** |
|  | N1.P1 - N1.P0 == 0 | 0.246774 | 0.342184 | 0.721 | 0.9794 | 0.47080 |
|  | N2.P1 - N1.P0 == 0 | 0.254513 | 0.341537 | 0.745 | 0.9762 | 0.45615 |
|  | N0.P1 - N2.P0 == 0 | 0.951784 | 0.338043 | 2.816 | **0.0549** | **0.00487** |
|  | N1.P1 - N2.P0 == 0 | 0.397505 | 0.344738 | 1.153 | 0.8589 | 0.24888 |
|  | N2.P1 - N2.P0 == 0 | 0.405244 | 0.343721 | 1.179 | 0.8470 | 0.23840 |
|  | N1.P1 - N0.P1 == 0 | -0.554279 | 0.331479 | -1.672 | 0.5501 | **0.09450** |
|  | N2.P1 - N0.P1 == 0 | -0.546540 | 0.331007 | -1.651 | 0.5641 | **0.09871** |
|  | N2.P1 - N1.P1 == 0 | 0.007739 | 0.335891 | 0.023 | 1.0000 | 0.98162 |
|  |  |  |  |  |  |  |
| *I. vera* (without outlier) | N1.P0 - N0.P0 == 0 | -0.009473 | 0.324573 | -0.029 | 1.0000 | 0.97672 |
|  | N2.P0 - N0.P0 == 0 | -0.181412 | 0.328721 | -0.552 | 0.9939 | 0.58104 |
|  | N0.P1 - N0.P0 == 0 | 0.808943 | 0.314989 | 2568 | 0.1051 | **0.01022** |
|  | N1.P1 - N0.P0 == 0 | -0.100348 | 0.331304 | -0.303 | 0.9997 | 0.76197 |
|  | N2.P1 - N0.P0 == 0 | 0.179945 | 0.321753 | 0.559 | 0.9935 | 0.57598 |
|  | N2.P0 - N1.P0 == 0 | -0.171940 | 0.325020 | -0.529 | 0.9950 | 0.59680 |
|  | N0.P1 - N1.P0 == 0 | 0.818415 | 0.311239 | 2.630 | **0.0902** | **0.00855** |
|  | N1.P1 - N1.P0 == 0 | -0.090876 | 0.329516 | -0.276 | 0.9998 | 0.78271 |
|  | N2.P1 - N1.P0 == 0 | 0.189417 | 0.320101 | 0.592 | 0.9916 | 0.55402 |
|  | N0.P1 - N2.P0 == 0 | 0.990355 | 0.314663 | 3.147 | **0.0204** | **0.00165** |
|  | N1.P1 - N2.P0 == 0 | 0.081064 | 0.332601 | 0.244 | 0.9999 | 0.80744 |
|  | N2.P1 - N2.P0 == 0 | 0.361357 | 0.322479 | 1.121 | 0.8729 | 0.26247 |
|  | N1.P1 - N0.P1 == 0 | -0.909291 | 0.319976 | -2.842 | **0.0511** | **0.00449** |
|  | N2.P1 - N0.P1 == 0 | -0.628998 | 0.309602 | -2.032 | 0.3242 | **0.04219** |
|  | N2.P1 - N1.P1 == 0 | 0.280293 | 0.326370 | 0.859 | 0.9561 | 0.39044 |
|  |  |  |  |  |  |  |
| *S. terebinthifolia* | N1.P0 - N0.P0 == 0 | -0.42400 | 0.42527 | -0.997 | 0.919 | 0.3188 |
|  | N2.P0 - N0.P0 == 0 | 0.20556 | 0.41692 | 0.493 | 0.996 | 0.6220 |
|  | N0.P1 - N0.P0 == 0 | -0.06857 | 0.40579 | -0.169 | 1.000 | 0.8658 |
|  | N1.P1 - N0.P0 == 0 | 0.58568 | 0.40245 | 1.455 | 0.693 | 0.1456 |
|  | N2.P1 - N0.P0 == 0 | -0.37488 | 0.43038 | -0.871 | 0.953 | 0.3837 |
|  | N2.P0 - N1.P0 == 0 | 0.62955 | 0.43028 | 1.463 | 0.688 | 0.1434 |
|  | N0.P1 - N1.P0 == 0 | 0.35542 | 0.42511 | 0.836 | 0.961 | 0.4031 |
|  | N1.P1 - N1.P0 == 0 | 1.00967 | 0.41865 | 2.412 | 0.152 | **0.0159** |
|  | N2.P1 - N1.P0 == 0 | 0.04912 | 0.44365 | 0.111 | 1.000 | 0.9118 |
|  | N0.P1 - N2.P0 == 0 | -0.27413 | 0.41599 | -0.659 | 0.986 | 0.5099 |
|  | N1.P1 - N2.P0 == 0 | 0.38012 | 0.40889 | 0.930 | 0.939 | 0.3526 |
|  | N2.P1 - N2.P0 == 0 | -0.58043 | 0.42792 | -1.356 | 0.753 | 0.1750 |
|  | N1.P1 - N0.P1 == 0 | 0.65425 | 0.40238 | 1.626 | 0.581 | 0.1040 |
|  | N2.P1 - N0.P1 == 0 | -0.30630 | 0.42896 | -0.714 | 0.980 | 0.4752 |
|  | N2.P1 - N1.P1 == 0 | -0.96055 | 0.42034 | -2.285 | 0.200 | **0.0223** |
|  |  |  |  |  |  |  |
| *S. lycocarpum* (with outlier) | N1.P0 - N0.P0 == 0 | 1.4007 | 0.6897 | 2.031 | 0.3220 | **0.04227** |
|  | N2.P0 - N0.P0 == 0 | 0.6846 | 0.6931 | 0.988 | 0.9211 | 0.32329 |
|  | N0.P1 - N0.P0 == 0 | 0.9423 | 0.7219 | 1.305 | 0.7803 | 0.19181 |
|  | N1.P1 - N0.P0 == 0 | 0.2745 | 0.7255 | 0.378 | 0.9990 | 0.70515 |
|  | N2.P1 - N0.P0 == 0 | -0.4754 | 0.7725 | -0.615 | 0.9898 | 0.53825 |
|  | N2.P0 - N1.P0 == 0 | -0.7161 | 0.5751 | -1.245 | 0.8127 | 0.21309 |
|  | N0.P1 - N1.P0 == 0 | -0.4584 | 0.6132 | -0.747 | 0.9756 | 0.45478 |
|  | N1.P1 - N1.P0 == 0 | -1.1262 | 0.6133 | -1.836 | 0.4393 | **0.06631** |
|  | N2.P1 - N1.P0 == 0 | -1.8761 | 0.6531 | -2.872 | **0.0462** | **0.00407** |
|  | N0.P1 - N2.P0 == 0 | 0.2577 | 0.6217 | 0.415 | 0.9984 | 0.67844 |
|  | N1.P1 - N2.P0 == 0 | -0.4101 | 0.5984 | -0.685 | 0.9834 | 0.49317 |
|  | N2.P1 - N2.P0 == 0 | -1.1600 | 0.6572 | -1.765 | 0.4857 | **0.07757** |
|  | N1.P1 - N0.P1 == 0 | -0.6678 | 0.6605 | -1.011 | 0.9135 | 0.31202 |
|  | N2.P1 - N0.P1 == 0 | -1.4178 | 0.7070 | -2.005 | 0.3364 | **0.04493** |
|  | N2.P1 - N1.P1 == 0 | -0.7500 | 0.6981 | -1.074 | 0.8905 | 0.28267 |
|  |  |  |  |  |  |  |
| *S. lycocarpum* (without outlier) | N1.P0 - N0.P0 == 0 | 1.00788 | 0.66080 | 1.525 | 0.645 | 0.1272 |
|  | N2.P0 - N0.P0 == 0 | 0.67749 | 0.64204 | 1.055 | 0.898 | 0.2913 |
|  | N0.P1 - N0.P0 == 0 | 0.92032 | 0.67105 | 1.371 | 0.742 | 0.1702 |
|  | N1.P1 - N0.P0 == 0 | 0.24967 | 0.67690 | 0.369 | 0.999 | 0.7122 |
|  | N2.P1 - N0.P0 == 0 | -0.40535 | 0.71875 | -0.564 | 0.993 | 0.5728 |
|  | N2.P0 - N1.P0 == 0 | -0.33039 | 0.54987 | -0.601 | 0.991 | 0.5479 |
|  | N0.P1 - N1.P0 == 0 | -0.08757 | 0.58785 | -0.149 | 1.000 | 0.8816 |
|  | N1.P1 - N1.P0 == 0 | -0.75822 | 0.59090 | -1.283 | 0.793 | 0.1994 |
|  | N2.P1 - N1.P0 == 0 | -141.323 | 0.62587 | -2.258 | 0.210 | **0.0239** |
|  | N0.P1 - N2.P0 == 0 | 0.24282 | 0.57195 | 0.425 | 0.998 | 0.6712 |
|  | N1.P1 - N2.P0 == 0 | -0.42782 | 0.55257 | -0.774 | 0.972 | 0.4388 |
|  | N2.P1 - N2.P0 == 0 | -1.08284 | 0.60558 | -1.788 | 0.471 | **0.0738** |
|  | N1.P1 - N0.P1 == 0 | -0.67065 | 0.61370 | -1.093 | 0.883 | 0.2745 |
|  | N2.P1 - N0.P1 == 0 | -1.32566 | 0.65545 | -2.023 | 0.327 | **0.0431** |
|  | N2.P1 - N1.P1 == 0 | -0.65502 | 0.64942 | -1.009 | 0.914 | 0.3132 |
|  |  |  |  |  |  |  |
| *C. cf adamantium* | N1.P0 - N0.P0 == 0 | -0.8870 | 1.0230 | -0.867 | 0.952 | 0.3859 |
|  | N2.P0 - N0.P0 == 0 | -0.1376 | 0.8840 | -0.156 | 1.000 | 0.8763 |
|  | N0.P1 - N0.P0 == 0 | -0.6848 | 1.0757 | -0.637 | 0.988 | 0.5244 |
|  | N1.P1 - N0.P0 == 0 | -1.1488 | 1.0811 | -1.063 | 0.891 | 0.2879 |
|  | N2.P1 - N0.P0 == 0 | 1.0793 | 0.7942 | 1.359 | 0.743 | 0.1742 |
|  | N2.P0 - N1.P0 == 0 | 0.7494 | 0.9198 | 0.815 | 0.963 | 0.4152 |
|  | N0.P1 - N1.P0 == 0 | 0.2022 | 1.1576 | 0.175 | 1.000 | 0.8613 |
|  | N1.P1 - N1.P0 == 0 | -0.2618 | 1.1100 | -0.236 | 1.000 | 0.8136 |
|  | N2.P1 - N1.P0 == 0 | 1.9663 | 0.8746 | 2.248 | 0.208 | **0.0246** |
|  | N0.P1 - N2.P0 == 0 | -0.5472 | 1.0291 | -0.532 | 0.995 | 0.5949 |
|  | N1.P1 - N2.P0 == 0 | -1.0111 | 0.9462 | -1.069 | 0.889 | 0.2853 |
|  | N2.P1 - N2.P0 == 0 | 1.2170 | 0.6729 | 1.809 | 0.449 | **0.0705** |
|  | N1.P1 - N0.P1 == 0 | -0.4640 | 1.2051 | -0.385 | 0.999 | 0.7002 |
|  | N2.P1 - N0.P1 == 0 | 1.7641 | 0.9458 | 1.865 | 0.413 | **0.0622** |
|  | N2.P1 - N1.P1 == 0 | 2.2281 | 0.9233 | 2.413 | 0.145 | **0.0158** |
|  |  |  |  |  |  |  |
| *P. hians* | N1.P0 - N0.P0 == 0 | 2.85101 | 1.48793 | 1.916 | 0.369 | **0.0554** |
|  | N2.P0 - N0.P0 == 0 | 3.56809 | 1.43939 | 2.479 | 0.119 | **0.0132** |
|  | N0.P1 - N0.P0 == 0 | 1.46755 | 1.63180 | 0.899 | 0.941 | 0.3685 |
|  | N1.P1 - N0.P0 == 0 | 1.37090 | 1.67713 | 0.817 | 0.960 | 0.4137 |
|  | N2.P1 - N0.P0 == 0 | 0.51329 | 1.91384 | 0.268 | 1.000 | 0.7885 |
|  | N2.P0 - N1.P0 == 0 | 0.71709 | 0.69945 | 1.025 | 0.901 | 0.3053 |
|  | N0.P1 - N1.P0 == 0 | -1.38346 | 1.02947 | -1.344 | 0.742 | 0.1790 |
|  | N1.P1 - N1.P0 == 0 | -1.48011 | 1.09215 | -1.355 | 0.735 | 0.1753 |
|  | N2.P1 - N1.P0 == 0 | -2.33772 | 1.39686 | -1.674 | 0.525 | **0.0942** |
|  | N0.P1 - N2.P0 == 0 | -2.10055 | 0.97740 | -2.149 | 0.243 | **0.0316** |
|  | N1.P1 - N2.P0 == 0 | -2.19719 | 1.04027 | -2.112 | 0.261 | **0.0347** |
|  | N2.P1 - N2.P0 == 0 | -3.05481 | 1.36162 | -2.244 | 0.201 | **0.0249** |
|  | N1.P1 - N0.P1 == 0 | '-0.09665 | 1.27262 | -0.076 | 1.000 | 0.9395 |
|  | N2.P1 - N0.P1 == 0 | -0.95426 | 1.51684 | -0.629 | 0.988 | 0.5293 |
|  | N2.P1 - N1.P1 == 0 | -0.85761 | 1.57866 | -0.543 | 0.994 | 0.5870 |
| **Phytophagous mites density (assuming mites with indeterminate feeding habits are also phytophagous)** | | | | | |  |
| *I. vera* (with outlier) | N1.P0 - N0.P0 == 0 | 0.05733 | 0.30478 | 0.188 | 1.0000 | 0.85081 |
|  | N2.P0 - N0.P0 == 0 | -0.19607 | 0.30845 | -0.636 | 0.9883 | 0.52500 |
|  | N0.P1 - N0.P0 == 0 | 0.72500 | 0.29495 | 2.458 | 0.1366 | **0.01397** |
|  | N1.P1 - N0.P0 == 0 | 0.18111 | 0.30385 | 0.596 | 0.9913 | 0.55115 |
|  | N2.P1 - N0.P0 == 0 | 0.16090 | 0.30386 | 0.530 | 0.9950 | 0.59646 |
|  | N2.P0 - N1.P0 == 0 | -0.25339 | 0.30775 | -0.823 | 0.9633 | 0.41029 |
|  | N0.P1 - N1.P0 == 0 | 0.66767 | 0.29523 | 2.262 | 0.2099 | **0.02373** |
|  | N1.P1 - N1.P0 == 0 | 0.12378 | 0.30263 | 0.409 | 0.9985 | 0.68252 |
|  | N2.P1 - N1.P0 == 0 | 0.10357 | 0.30150 | 0.344 | 0.9994 | 0.73121 |
|  | N0.P1 - N2.P0 == 0 | 0.92107 | 0.29762 | 3.095 | **0.0242** | **0.00197** |
|  | N1.P1 - N2.P0 == 0 | 0.37717 | 0.30574 | 1.234 | 0.8204 | 0.21733 |
|  | N2.P1 - N2.P0 == 0 | 0.35696 | 0.30783 | 1.160 | 0.8559 | 0.24621 |
|  | N1.P1 - N0.P1 == 0 | -0.54389 | 0.29276 | -1.858 | 0.4284 | **0.06320** |
|  | N2.P1 - N0.P1 == 0 | -0.56410 | 0.29715 | -1.898 | 0.4028 | **0.05765** |
|  | N2.P1 - N1.P1 == 0 | -0.02021 | 0.30216 | -0.067 | 1.0000 | 0.94667 |
|  |  |  |  |  |  |  |
| *I. vera* (without outlier) | N1.P0 - N0.P0 == 0 | 0.08239 | 0.28139 | 0.293 | 0.9997 | 0.769667 |
|  | N2.P0 - N0.P0 == 0 | -0.17096 | 0.28579 | -0.598 | 0.9912 | 0.549712 |
|  | N0.P1 - N0.P0 == 0 | 0.77001 | 0.27058 | 2.846 | **0.0505** | **0.004430** |
|  | N1.P1 - N0.P0 == 0 | -0.07949 | 0.28784 | -0.276 | 0.9998 | 0.782424 |
|  | N2.P1 - N0.P0 == 0 | 0.17428 | 0.28065 | 0.621 | 0.9895 | 0.534620 |
|  | N2.P0 - N1.P0 == 0 | -0.25335 | 0.28354 | -0.894 | 0.9481 | 0.371576 |
|  | N0.P1 - N1.P0 == 0 | 0.68762 | 0.26875 | 2.559 | 0.1075 | **0.010510** |
|  | N1.P1 - N1.P0 == 0 | -0.16188 | 0.28627 | -0.565 | 0.9932 | 0.571740 |
|  | N2.P1 - N1.P0 == 0 | 0.09188 | 0.27714 | 0.332 | 0.9995 | 0.740230 |
|  | N0.P1 - N2.P0 == 0 | 0.94097 | 0.27218 | 3.457 | **0.0072** | **0.000546** |
|  | N1.P1 - N2.P0 == 0 | 0.09146 | 0.29050 | 0.315 | 0.9996 | 0.752874 |
|  | N2.P1 - N2.P0 == 0 | 0.34523 | 0.28394 | 1.216 | 0.8291 | 0.224036 |
|  | N1.P1 - N0.P1 == 0 | -0.84950 | 0.27750 | -3.061 | **0.026** | **0.002204** |
|  | N2.P1 - N0.P1 == 0 | -0.59573 | 0.27076 | -2.200 | 0.2371 | **0.027788** |
|  | N2.P1 - N1.P1 == 0 | 0.25377 | 0.28494 | 0.891 | 0.9488 | 0.373150 |
|  |  |  |  |  |  |  |
| *S. terebinthifolia* | N1.P0 - N0.P0 == 0 | -0.36264 | 0.38360 | -0.945 | 0.935 | 0.3445 |
|  | N2.P0 - N0.P0 == 0 | 0.17234 | 0.37587 | 0.459 | 0.997 | 0.6466 |
|  | N0.P1 - N0.P0 == 0 | -0.14121 | 0.36944 | -0.382 | 0.999 | 0.7023 |
|  | N1.P1 - N0.P0 == 0 | 0.56625 | 0.36475 | 1.552 | 0.630 | 0.1206 |
|  | N2.P1 - N0.P0 == 0 | -0.21012 | 0.38211 | -0.550 | 0.994 | 0.5824 |
|  | N2.P0 - N1.P0 == 0 | 0.53499 | 0.38584 | 1.387 | 0.735 | 0.1656 |
|  | N0.P1 - N1.P0 == 0 | 0.22143 | 0.38436 | 0.576 | 0.993 | 0.5645 |
|  | N1.P1 - N1.P0 == 0 | 0.92889 | 0.37694 | 2.464 | 0.135 | **0.0137** |
|  | N2.P1 - N1.P0 == 0 | 0.15252 | 0.39208 | 0.389 | 0.999 | 0.6973 |
|  | N0.P1 - N2.P0 == 0 | -0.31356 | 0.37629 | -0.833 | 0.961 | 0.4047 |
|  | N1.P1 - N2.P0 == 0 | 0.39390 | 0.36805 | 1.070 | 0.893 | 0.2845 |
|  | N2.P1 - N2.P0 == 0 | -0.38246 | 0.37910 | -1.009 | 0.915 | 0.3130 |
|  | N1.P1 - N0.P1 == 0 | 0.70746 | 0.36642 | 1.931 | 0.383 | **0.0535** |
|  | N2.P1 - N0.P1 == 0 | -0.06891 | 0.38179 | -0.180 | 1.000 | 0.8568 |
|  | N2.P1 - N1.P1 == 0 | -0.77636 | 0.37277 | -2.083 | 0.296 | **0.0373** |
|  |  |  |  |  |  |  |
| *C. cf adamantium* (with outlier) | N1.P0 - N0.P0 == 0 | -0.4775 | 0.7167 | -0.666 | 0.9851 | 0.50524 |
|  | N2.P0 - N0.P0 == 0 | -0.2278 | 0.7047 | -0.323 | 0.9995 | 0.74649 |
|  | N0.P1 - N0.P0 == 0 | -0.7935 | 0.7731 | -1.026 | 0.9069 | 0.30473 |
|  | N1.P1 - N0.P0 == 0 | -1.4085 | 0.8485 | -1.660 | 0.5520 | **0.09689** |
|  | N2.P1 - N0.P0 == 0 | 0.6307 | 0.6431 | 0.981 | 0.9222 | 0.32675 |
|  | N2.P0 - N1.P0 == 0 | 0.2497 | 0.6637 | 0.376 | 0.9990 | 0.70671 |
|  | N0.P1 - N1.P0 == 0 | -0.3160 | 0.7664 | -0.412 | 0.9984 | 0.68014 |
|  | N1.P1 - N1.P0 == 0 | -0.9310 | 0.8187 | -1.137 | 0.8627 | 0.25549 |
|  | N2.P1 - N1.P0 == 0 | 1.1082 | 0.6184 | 1.792 | 0.4642 | **0.07311** |
|  | N0.P1 - N2.P0 == 0 | -0.5657 | 0.7303 | -0.775 | 0.9710 | 0.43855 |
|  | N1.P1 - N2.P0 == 0 | -1.1807 | 0.7707 | -1.532 | 0.6374 | 0.12551 |
|  | N2.P1 - N2.P0 == 0 | 0.8585 | 0.5636 | 1.523 | 0.6432 | 0.12767 |
|  | N1.P1 - N0.P1 == 0 | -0.6150 | 0.8750 | -0.703 | 0.9811 | 0.48211 |
|  | N2.P1 - N0.P1 == 0 | 1.4242 | 0.6859 | 2.077 | 0.2938 | **0.03785** |
|  | N2.P1 - N1.P1 == 0 | 2.0392 | 0.7433 | 2.744 | **0.0651** | **0.00608** |
|  |  |  |  |  |  |  |
| *C. cf adamantium* (without outlier) | N1.P0 - N0.P0 == 0 | 0.29350 | 0.71271 | 0.412 | 0.9984 | 0.68048 |
|  | N2.P0 - N0.P0 == 0 | 0.27730 | 0.68170 | 0.407 | 0.9985 | 0.68417 |
|  | N0.P1 - N0.P0 == 0 | -0.04148 | 0.76016 | -0.055 | 10000 | 0.95648 |
|  | N1.P1 - N0.P0 == 0 | -0.77707 | 0.85501 | -0.909 | 0.9418 | 0.36344 |
|  | N2.P1 - N0.P0 == 0 | 1.46578 | 0.61969 | 2.365 | 0.1623 | **0.01801** |
|  | N2.P0 - N1.P0 == 0 | -0.01620 | 0.60731 | -0.027 | 1.0000 | 0.97871 |
|  | N0.P1 - N1.P0 == 0 | -0.33499 | 0.70951 | -0.472 | 0.9969 | 0.63683 |
|  | N1.P1 - N1.P0 == 0 | -1.07057 | 0.78921 | -1.357 | 0.7446 | 0.17494 |
|  | N2.P1 - N1.P0 == 0 | 1.17227 | 0.54185 | 2.163 | 0.2465 | **0.03050** |
|  | N0.P1 - N2.P0 == 0 | -0.31878 | 0.68407 | -0.466 | 0.9971 | 0.64121 |
|  | N1.P1 - N2.P0 == 0 | -1.05437 | 0.74544 | -1.414 | 0.7093 | 0.15724 |
|  | N2.P1 - N2.P0 == 0 | 1.18848 | 0.49983 | 2.378 | 0.1577 | **0.01742** |
|  | N1.P1 - N0.P1 == 0 | -0.73558 | 0.85745 | -0.858 | 0.9542 | 0.39096 |
|  | N2.P1 - N0.P1 == 0 | 1.50726 | 0.61858 | 2.437 | 0.1381 | **0.01482** |
|  | N2.P1 - N1.P1 == 0 | 2.24284 | 0.71664 | 3.130 | **0.0206** | **0.00175** |
|  |  |  |  |  |  |  |
| *P. hians* | N1.P0 - N0.P0 == 0 | 2.8744 | 1.4215 | 2.022 | 0.3079 | **0.04317** |
|  | N2.P0 - N0.P0 == 0 | 3.5981 | 1.3756 | 2.616 | **0.0844** | **0.00891** |
|  | N0.P1 - N0.P0 == 0 | 1.4658 | 1.5598 | 0.940 | 0.9295 | 0.34735 |
|  | N1.P1 - N0.P0 == 0 | 1.9684 | 1.5293 | 1.287 | 0.7754 | 0.19805 |
|  | N2.P1 - N0.P0 == 0 | 0.5056 | 1.8277 | 0.277 | 0.9998 | 0.78205 |
|  | N2.P0 - N1.P0 == 0 | 0.7238 | 0.6622 | 1.093 | 0.8734 | 0.27439 |
|  | N0.P1 - N1.P0 == 0 | -1.4086 | 0.9802 | -1.437 | 0.6836 | 0.15072 |
|  | N1.P1 - N1.P0 == 0 | -0.9060 | 0.9076 | -0.998 | 0.9104 | 0.31818 |
|  | N2.P1 - N1.P0 == 0 | -2.3688 | 1.3294 | -1.782 | 0.4533 | **0.07478** |
|  | N0.P1 - N2.P0 == 0 | -2.1323 | 0.9291 | -2.295 | 0.1800 | **0.02173** |
|  | N1.P1 - N2.P0 == 0 | -1.6297 | 0.8545 | -1.907 | 0.3741 | **0.05648** |
|  | N2.P1 - N2.P0 == 0 | -3.0925 | 1.2943 | -2.389 | 0.1459 | **0.01687** |
|  | N1.P1 - N0.P1 == 0 | 0.5026 | 1.0960 | 0.459 | 0.9971 | 0.64652 |
|  | N2.P1 - N0.P1 == 0 | -0.9602 | 1.4480 | -0.663 | 0.9842 | 0.50728 |
|  | N2.P1 - N1.P1 == 0 | -1.4628 | 1.4036 | -1.042 | 0.8942 | 0.29733 |
| **Predators mites occurrence** |  |  |  |  |  |  |
| *I. vera* | N1.P0 - N0.P0 == 0 | -0.5365 | 0.4612 | -1.163 | 0.854 | 0.2446 |
|  | N2.P0 - N0.P0 == 0 | -0.3654 | 0.4590 | -0.796 | 0.968 | 0.4259 |
|  | N0.P1 - N0.P0 == 0 | -0.6629 | 0.4700 | -1.410 | 0.721 | 0.1584 |
|  | N1.P1 - N0.P0 == 0 | 0.4177 | 0.4727 | 0.884 | 0.951 | 0.3769 |
|  | N2.P1 - N0.P0 == 0 | -0.1867 | 0.4653 | -0.401 | 0.999 | 0.6882 |
|  | N2.P0 - N1.P0 == 0 | 0.1711 | 0.4580 | 0.374 | 0.999 | 0.7086 |
|  | N0.P1 - N1.P0 == 0 | -0.1264 | 0.4692 | -0.269 | 1.000 | 0.7876 |
|  | N1.P1 - N1.P0 == 0 | 0.9542 | 0.4733 | 2.016 | 0.333 | **0.0438** |
|  | N2.P1 - N1.P0 == 0 | 0.3499 | 0.4652 | 0.752 | 0.975 | 0.4520 |
|  | N0.P1 - N2.P0 == 0 | -0.2975 | 0.4673 | -0.637 | 0.988 | 0.5244 |
|  | N1.P1 - N2.P0 == 0 | 0.7831 | 0.4716 | 1.660 | 0.558 | **0.0969** |
|  | N2.P1 - N2.P0 == 0 | 0.1787 | 0.4628 | 0.386 | 0.999 | 0.6994 |
|  | N1.P1 - N0.P1 == 0 | 1.0806 | 0.4822 | 2.241 | 0.219 | **0.0250** |
|  | N2.P1 - N0.P1 == 0 | 0.4762 | 0.4744 | 1.004 | 0.917 | 0.3154 |
|  | N2.P1 - N1.P1 == 0 | -0.6044 | 0.4767 | -1.268 | 0.803 | 0.2048 |
|  |  |  |  |  |  |  |

**Table S6.** Percentage (%) of zeros detected for the different response variables of herbivory and predation.

| % of zeros in the response variable | *I. vera*  (Fabaceae) | *S. terebinthifolia* (Anacardiaceae) | *S. lycocarpum* (Solanaceae) | *C. cf adamantium* (Myrtaceae) | *P. hians* (Myrtaceae) | *E. gemmiflora* (Myrtaceae) |
| --- | --- | --- | --- | --- | --- | --- |
| Leaf herbivory (%) | 3% | 42% | 28% | 54% | 13% | 56% |
| Phytophagous mites density | 22% | 55% | 73% | 86% | 77% | 98% |
| Phytophagous mites density (assuming mites with indeterminate feeding habits are also phytophagous) | 16% | 46% | 63% | 75% | 73% | 91% |
| Predators mites occurrence | 50% | 72% | 77% | 80% | 88% | 93% |


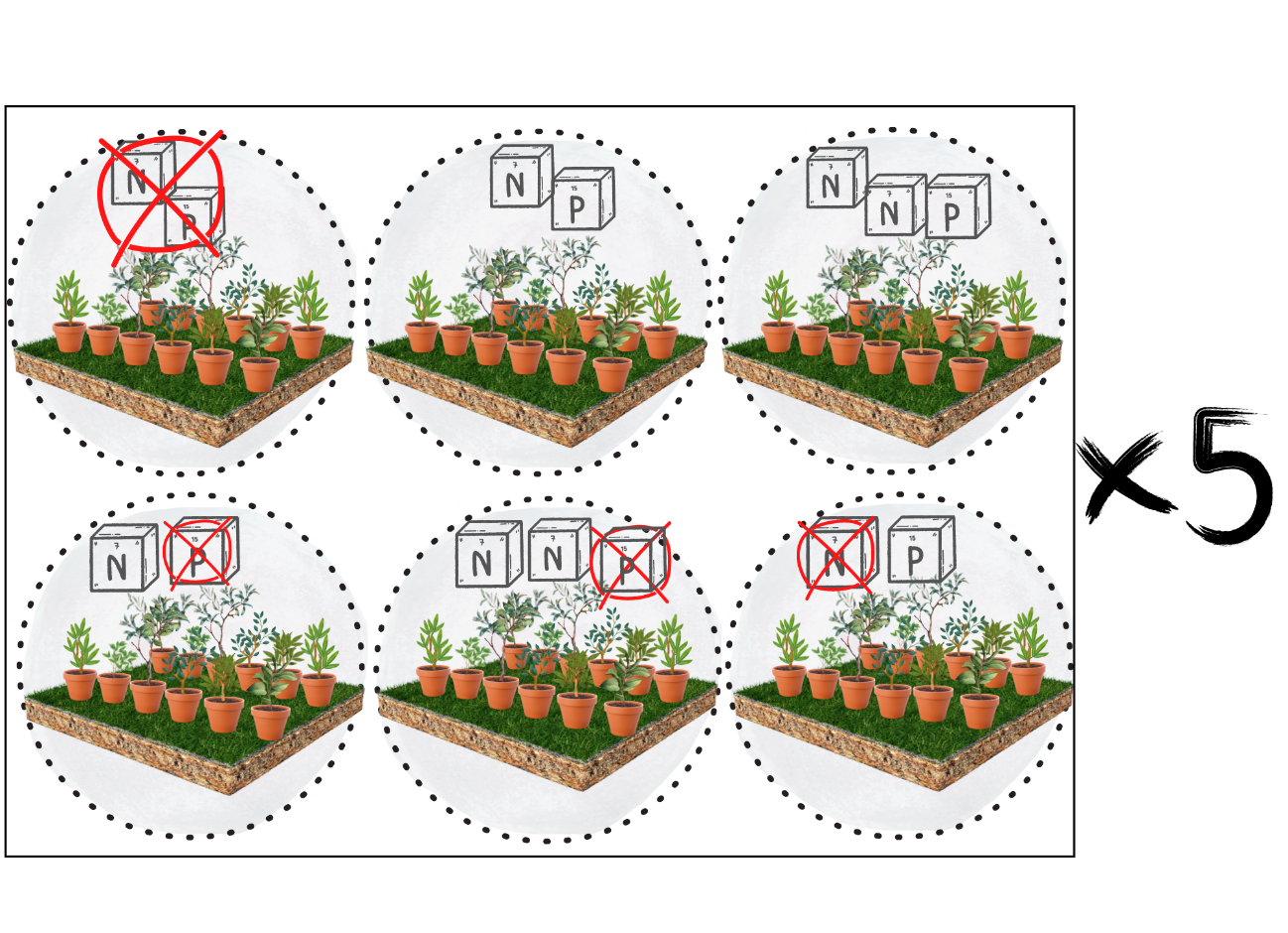


**Figure S1.** Experimental design consisting of five study sites (solid line) each containing six treatments (dashed line). Each treatment is formed by a grouping of plants of six studied species. The pots were positioned next to each other forming two rows of pots per treatment with approximately 5 cm between pots in the same row and approximately 20 cm between rows. The distance between treatments was also approximately 20 cm.


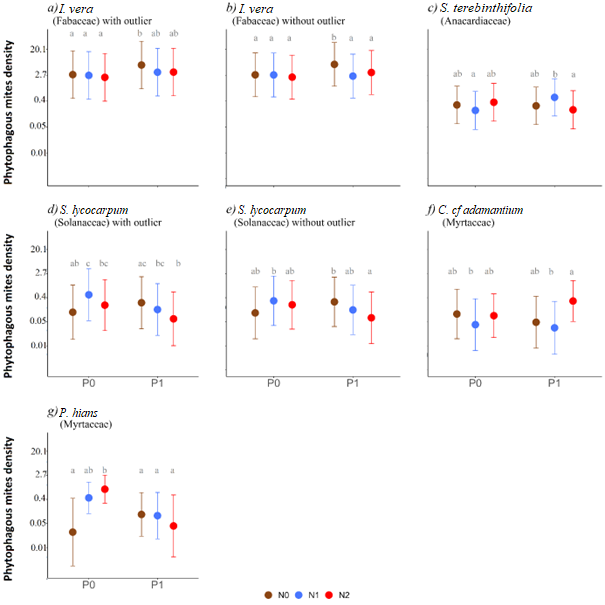


**Figure S2.** Effect of the fertilization treatments on phytophagous mite density (individuals per leaf), for each plant species. Mean estimates and associated 95% confidence intervals are shown. The y-axis presents back-transformed values: all estimates originally modeled on a logarithmic scale were converted back to their corresponding exponential units to facilitate interpretation. N0 = no N addition, N1 = 60 kg N/ha, N2 = 130 kg N/ha; P0 = no P addition, P1 = 40 kg P/ha. Whenever the probability of N or P having a significant effect exceeded 90% (P-value < 0.1; Table S4), post hoc tests were done to compare values obtained under different nutrient combinations (values that have a probability of being different higher than 95%, i.e. P<0.05, are indicated with distinct letters). Details of the statistical analyses are provided in Tables S4 and S5.

####
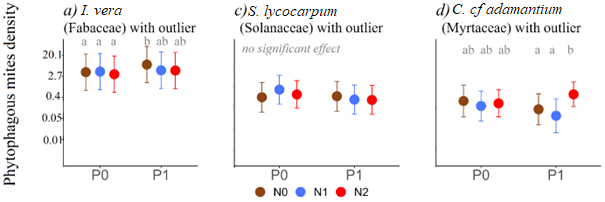


**Figure S3.** Effect of the fertilization treatments on phytophagous mite density (individuals per leaf), assuming mites with indeterminate feeding habits are also phytophagous, detected on each plant species. Mean estimates and associated 95% confidence intervals are shown. The y-axis presents back-transformed values: all estimates originally modeled on a logarithmic scale were converted back to their corresponding exponential units to facilitate interpretation. N0 = no N addition, N1 = 60 kg N/ha, N2 = 130 kg N/ha; P0 = no P addition, P1 = 40 kg P/ha. Whenever the probability of N or P having a significant effect exceeded 90% (P-value < 0.1; Table S4), post hoc tests were done to compare values obtained under different nutrient combinations (values that have a probability of being different higher than 95%, i.e. P<0.05, are indicated with distinct letters). Details of the statistical analyses are provided in Tables S4 and S5.

#### References

#### Almeida, C. D. (2022). What is planted in the restoration of the Atlantic Forest: floristic and functional analysis (Doctoral dissertation, Universidade de São Paulo).

Bezerra, J. E. F., daSilva-Junior, J. F., & de Lira-Junior, J. S. (2018). Psidium guineese: araça. Embrapa Tabuleiros Costeiros. pp. 270-278. In: Coradin, L.; Camillo, J.; Pareyn, F. G. C. (Ed.). Espécies nativas da flora brasileira de valor econômico atual ou potencial: plantas para o futuro: região Nordeste. Brasília, DF: MMA.

Carvalho, P. (2003). Espécies arbóreas brasileiras Aroeira-Pimenteira: *Schinus terebinthifolius*. (Volume 1).

Carvalho, P. (2008). Espécies arbóreas brasileiras. Ingá-banana: *Inga vera* subsp. affinis. (Volume 3)

Carvalho, P. (2010). Espécies arbóreas brasileiras. Lobeira: *Solanum lycocarpum*. (Volume 4).

deAlmeida, C., & Viani, R. A. G. (2021). Non-continuous reproductive phenology of animal-dispersed species in young forest restoration plantings. Biotropica, 53(1), 266–275. https://doi.org/10.1111/btp.12869

Guedes, J. dos S., & Krupek, R. A. (2017). Ecological characteristics and phytosanitary of tree species in a Rain Forest fragment of southeast region of São Paulo state. Ambiência, 13(2), 311–324. https://doi.org/10.5935/ambiencia.2017.02.04

Hothorn, T., Bretz, F., Westfall, P., Heiberger, R. M., Schuetzenmeister, A., Scheibe, S., & Hothorn, M. T. (2016). Package ‘multcomp’, Simultaneous inference in general parametric models. Project for Statistical Computing, Vienna, Austria, 1-36.

Kuhlmann, M. & Fagg, C. (2018). Frutos e sementes do Cerrado: espécies atrativas para fauna - Volume I e II-2.ed.

Lorenzi, H. (2016). Árvores brasileiras: manual de identificação e cultivo de plantas arbóreas nativas do Brasil. v.1.7a ed. Editora Plantarum, Nova Odessa.

Oliveira, S. C. C., Ferreira, A. G., & Borghetti, F. (2004). Efeito alelopático de folhas de Solanum lycocarpum A. St.-Hil. (Solanaceae) na germinação e crescimento de Sesamum indicum L. (Pedaliaceae) sob diferentes temperaturas. Acta Botanica Brasilica, 18(3), 401–406. https://doi.org/10.1590/S0102-33062004000300001

Isernhagen, I. (2015). Listagem florística de espécies arbóreas e arbustivas de Mato Grosso: um ponto de partida para projetos de restauração ecológica. Embrapa Agrossilvipastoril Sinop, MT
